# The USP11/Nrf2 positive feedback loop promotes colorectal cancer progression by inhibiting mitochondrial apoptosis

**DOI:** 10.1038/s41419-024-07188-2

**Published:** 2024-12-01

**Authors:** Yuanyuan Lu, Wanhui wei, Mengting Li, Danyang Chen, Wenjie Li, Qian Hu, Shouquan Dong, Lan Liu, Qiu Zhao

**Affiliations:** 1 Department of Gastroenterology, Zhongnan Hospital of Wuhan University, Wuhan, China; 2 Hubei Clinical Center and Key Lab of Intestinal and Colorectal Diseases, Wuhan, China; 3 Department of Gastroenterology, Wuhan Third Hospital, Tongren Hospital of Wuhan University, Wuhan, China; 4 Department of Gastroenterology, The Central Hospital of Wuhan, Tongji Medical College, Huazhong University of Science and Technology, Wuhan, China; 5 Key Laboratory for Molecular Diagnosis of Hubei Province, The Central Hospital of Wuhan, Tongji Medical College, Huazhong University of Science and Technology, Wuhan, China; 6 Department of Gastroenterology, Wuhan Children’s Hospital, Tongji Medical College, Huazhong University of Science and Technology, Wuhan, China; 7 Department of Hematology, West China Hospital, Sichuan University, Chengdu, China

**Keywords:** Gastrointestinal diseases, Colorectal cancer

## Abstract

Abnormal antioxidant capacity of cancer is closely related to tumor malignancy. Modulation of oxidative stress status is a novel anticancer therapeutic target. Nrf2 is a key regulator of various antioxidant enzymes, but the mechanism of its deubiquitination remains largely unclear. This study unveiled that Nrf2 received post-transcriptional regulation from a proteasome-associated deubiquitinating enzyme, USP11, in colorectal cancer (CRC). It was found that USP11 was overexpressed in CRC tissues acting as an oncogene by inhibiting mitochondrial apoptosis, and USP11 managed to maintain balance in the production and elimination of reactive oxygen species (ROS). Mechanistically, we identified a feedback loop between USP11 and Nrf2 maintaining the redox homeostasis. USP11 stabilized Nrf2 by deubiquitinating and protecting it from proteasome-mediated degradation. Interestingly, we also map that Nrf2 could bind to the antioxidant reaction element (ARE) in the USP11 promoter to promote its transcription. Hence, USP11/Nrf2 positive feedback loop inhibited mitochondrial apoptosis of CRC cells by activating Nrf2/ARE signaling pathway, thus promoting CRC progression.

**Figure:**
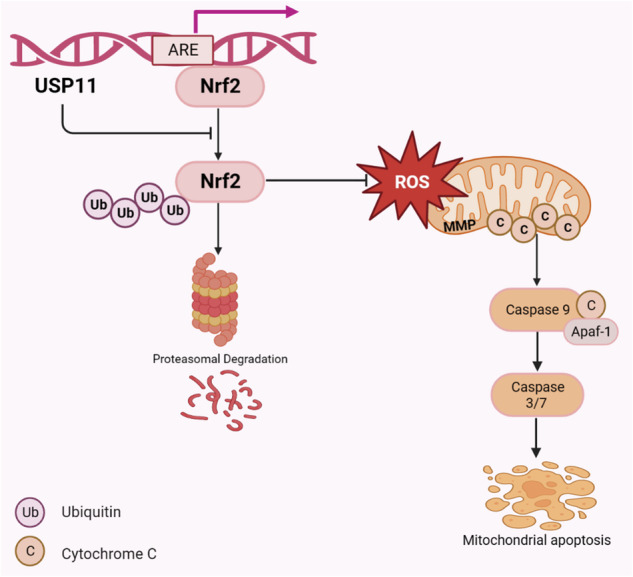
*Schematic diagram of the mechanism by which USP11/Nrf2 positive feedback loop inhibited mitochondrial apoptosis in CRC cells*. This study found that USP11 was highly expressed in colorectal cancer (CRC) tissue and was associated with poor prognosis. In CRC, the inhibition of USP11 expression could promote the ubiquitination degradation of Nrf2, thereby inhibiting the Nrf2/ARE signaling pathway. This led to an increase in reactive oxygen species in the cell, causing mitochondrial apoptosis. In addition, Nrf2 could bind to the promoter region of USP11 to promote its transcription, both of which formed positive feedback loop.

*Schematic diagram of the mechanism by which USP11/Nrf2 positive feedback loop inhibited mitochondrial apoptosis in CRC cells*. This study found that USP11 was highly expressed in colorectal cancer (CRC) tissue and was associated with poor prognosis. In CRC, the inhibition of USP11 expression could promote the ubiquitination degradation of Nrf2, thereby inhibiting the Nrf2/ARE signaling pathway. This led to an increase in reactive oxygen species in the cell, causing mitochondrial apoptosis. In addition, Nrf2 could bind to the promoter region of USP11 to promote its transcription, both of which formed positive feedback loop.

## Introduction

An alarming observation came to light with more than 1.9 million new cases of colorectal cancer (CRC) being diagnosed and 935,000 recorded deaths worldwide in the year. This disturbing data catapulted CRC to the third highest position in terms of incidence rates and second in fatality rates [1]. Given these exceedingly high rates of occurrence and fatality, CRC posed a significant threat to human health and called for urgent attention. Surgery, chemotherapy, radiotherapy, target therapy, and immunotherapy were the primary treatments for CRC, but their efficacy was limited and only beneficial to a subset of patients [2–4]. Thus, it was crucial to conduct further research to discover more effective treatments or enhance the efficiency of current therapies.

Reactive Oxygen Species (ROS), a principal molecule produced by oxidative stress, was considered to be a significant factor in the development, proliferation, and recurrence of tumors. However, extremely high ROS levels could also lead to apoptosis.

Thus, modulation of oxidative stress status was a novel anticancer therapeutic target. Nuclear factor erythroid 2-related factor 2 (Nrf2), also known as NFE2L2, was a key molecule of intracellular antioxidant. It identified and bound with antioxidant response elements (AREs), promoting downstream gene expression and regulating endogenous REDOX system balance [5]. ARE was a 5 ‘-TGACnnnGC-3’ sequence in the gene promoter region and was located in the promoter sequence of SOD, NQO1, and other antioxidant oxidase genes. Nowadays, a large number of literatures have confirmed that Nrf2 could be degraded through three ways of ubiquitination modification: KEAP1-CUL3-RBX1, β-TrCP-SKP1-CUL1-RBX1, and HRD1 [6–10], but the deubiquitination of Nrf2 remained to be further studied.

The ubiquitin-proteasome system was primarily responsible for protein degradation in eukaryotic cells. In this system, proteins are tagged with ubiquitin and sent to the 26S proteasome for disassembly. proteins were labeled with ubiquitination and transported to the 26S proteasome for degradation. However, this process could be reversed by deubiquitinating enzymes, which hydrolyzed ester bonds, peptide bonds, and isopeptide bonds at the carboxyl end of ubiquitin and detached the ubiquitin molecules from the target proteins. This played a critical role in the regulation of vital biological processes such as cell cycle, DNA repair, and cell apoptosis [11–13]. USP11, also known as UHX1, was a member of Ubiquitin-specific proteases (USPs). a series of studies revealed significant disparities regarding USP11 expression between tumor tissues and adjacent tissues. Its deubiquitinating activity regulated the stability of substrate proteins and was involved in the occurrence and development of tumors.

USP11 has been identified to bind to the C-terminal of BRCA2 (2281-3418) and stabilize BRCA2 protein through deubiquitination, thus participating in DNA damage repair of breast cancer [14]. Further, it has been observed that high expression of USP11 could deubiquitinate and stabilize Snail, thereby promoting the Epithelial-Mesenchymal Transition of ovarian cancer [15]. Moreover, aberrant USP11 expression could accelerate proliferation and migration in Hepatocellular carcinoma [16, 17]. Mitoxantrone, as an inhibitor of USP11, has already been demonstrated in several clinical applications, including the treatment of acute myeloid leukemia, hormone-refractory prostate cancer, and multiple sclerosis [18–20]. Recently, it has also been reported that mitoxantrone has been used in the treatment of pancreatic cancer [21]. As such, USP11 emerged as a promising biomarker in the context of cancer.

Despite Nrf2/ARE pathway and USP11 appeared to be critical roles in tumor, it remained unclear whether USP11 contributed to the regulation of Nrf2 in CRC. In this study, we investigated the effect of USP11 on apoptosis in CRC and its underlying molecular mechanism. Our results demonstrated that USP11 protected Nrf2 from proteasome degradation by removing the K48-linked polyubiquitin chain of Nrf2. In addition, transcription factor Nrf2 could promote USP11 transcription, both of which formed positive feedback loop that regulated mitochondrial apoptosis in CRC.

## Methods

### Colorectal cancer samples

10 CRC tissues and matched adjacent tissues were collected from Zhongnan Hospital of Wuhan University. The study was approved by the Ethics Committee of Zhongnan Hospital of Wuhan University and informed consent was obtained from all enrolled patients.

### Cell culture

Human CRC cell lines (Ncm460, HT29, HCT116, DLD1, CaCO2, SW480) were bought from National Collection of Authenticated Cell Cultures. Ncm460, HT29, CaCO2, and SW480 CRC cell lines were maintained in 1640 medium (Hyclone, USA) supplemented with 10% fetal bovine serum (ABW, China) and 1% penicillin-Streptomycin (Biosharp, China). According to the expression of USP11 in different CRC cell lines, HCT116/DLD1 cells were selected for further study (Fig. S2). HCT116 cell lines was maintained in McCoy’s 5 A medium (Procell, Wuhan, China) supplemented with 10% fetal bovine serum (ABW, China) and 1% penicillin-Streptomycin (Biosharp, China). DLD1 cell lines was maintained in DMEM medium (KeyGEN BioTECH, China) supplemented with 10% fetal bovine serum (ABW, China) and 1% penicillin-Streptomycin (Biosharp, China). All cells were cultured at maintained at 37 °C with 5% CO_2_.

### Quantitative Real-time PCR (qPCR)

Total RNA of cells was extracted by Trizol reagent (Invitrogen, California, USA) according to the instruction manual. cDNA was generated using qPCR RT kit (TOYOBO, Japan). qPCR was performed with UltraSYBR Green Mixture 2× (CWBIO, China). All primers were synthesized by the Tsingke Company (Wuhan), and the sequences of primers were shown in Table S1.

### Western blotting

Total protein from tissue samples or cells was extracted from Western and IP cell lysate (Beyotime Biotechnology, China). The protein concentration was quantified by BCA kits (Beyotime Biotechnology, China). Proteins were separated by SDS-PAGE and transferred to PVDF membranes (Millipore, USA). Then, the membrane was blocked with 5% fat-free milk and incubated with the following primary antibodies: USP11 (Abcam Ab109232, UK); Cleaved PARP1(Zenbio R23942, China); Procaspase9/Cleaved caspase9 (CST 9508, USA); Bcl2 (CST 3498, USA); Bak (CST 12105, USA); Bax (CST 2772, USA); Cleaved Caspase3 (CST 9664, USA); Cytochrome C (CST 11940, USA); Nrf2 (CST 12721, USA); HO-1(Abclonal Technology A11919, China); NQO1(Abcam Ab80588, UK); Flag-tag (CST 14793, USA);His-tag (CST 2366, USA) ;GAPDH (Proteintech 10494-1-AP, USA). The second day, the membrane was incubated with Anti-rabbit/mouse IgG-HRP (Servicebio GB23303/GB23301, China) secondary antibody, and Chemiluminescent signals were detected by Enhanced Chemiluminescence (Thermo, USA).

### Immunohistochemistry (IHC)

The IHC experiments were conducted according to standard procedures. Tissues were fixed with 4% paraformaldehyde, embedded in paraffin, sectioned, dewaxed, and antigen retrieval. What’s more, endogenous peroxidase was blocked. The slides were incubated with the following primary antibodies: USP11 (HuaBio ET1705-38, China); Nrf2; Ki67 (Servicebio GB121141-100, China). The next day, the slides were then incubated with HRP-conjugated anti-rabbit/mouse secondary antibody (Servicebio G1214-100UL/G1213-100UL, China), and Diaminobenzidine (DAB) was used as a chromogenic agent. IHC images were captured under white light by a fluorescence microscope (Olympus, Japan).

### In vitro assays

Plasmids and siRNA transfection

USP11 siRNAs, and corresponding negative controls were purchased from RiboBio (China). The Flag-USP11 and His-Nrf2 plasmids were obtained from Sino Biological (China). Lipofectamine 2000 transfection kit (Invitrogen, USA) was used for siRNAs and plasmids were transfected into the cells using Lipofectamine 3000 (Invitrogen, USA) following the manufacturer’s protocol. The siRNA sequences were provided in Table S2.

### Apoptosis assays

Apoptosis was measured by flow cytometry using the Annexin V-PI Apoptosis Detection Kits (Bestbio, China) according to the protocol in the manufacturer.

### CCK-8 cell proliferation assay

HCT116/DLD1 cells were seeded in 96-well plates at a density of 2000 cells per well and transfected with the designated siRNA. Cell proliferation rates were measured using the Cell Counting Kit-8 (CCK-8, Dojindo) at 0 h, 24 h, 48 h, 72 h and 96 h respectively. The optical density (OD) at a wavelength of 450 nm for each well was determined by a microplate reader.

### Mitochondrial membrane potential (MMP) measurement

HCT116/DLD1 cells were seeded in 6-well plates and transfected with the designated siRNA. Then cells were stained with JC-1 (Beyotime Biotechnology, China) and observed with fluorescence microscopy.

### Measurement of ATP levels

The ATP content in HCT116/DLD1 cells was detected using the Enhanced ATP Assay Kit (Beyotime Biotechnology, China) according to the protocol in the manufacturer.

### ROS assay

Fluorometric Intracellular ROS Kits (Bestbio, Invitrogen) were used to detect the ROS levels in HCT116/DLD1 cells according to the protocol in the manufacturer.

### GSH assay

The relative GSH concentration in HCT116/DLD1 cells was assessed using a kit from Solarbio (Solarbio, China) according to the protocol in the manufacturer.

### Co-immunoprecipitation (Co-IP) assay

For Co-IP assays, equal amounts of total protein were incubated with following primary antibodies at 4 °C overnight: USP11; Nrf2; Flag-tag (CST 14793, USA);His-tag (CST 2366,USA) ;IgG (CST, USA). On the second day, 20 μL of protein A/G PLUS-Agarose beads (Santa Cruz Biotechnology, USA) were added to each group and then incubated for 3 h. Precipitation was analyzed using standard Western blotting methods.

### Ubiquitination assay

HCT116/DLD1 cells were infected with siNC or siUSP11 for 48 h, followed by treatment with 20 μM MG132 (MedChemExpress HY-13259, USA) for 6 h. Co-IP analysis was then performed.

### Cycloheximide (CHX) and MG132 treatment assays

HCT116/DLD1 cells were infected with siNC or siUSP11 for 48 h and treated with MG132 (20 μM) for 6 h or CHX (50 μg/ml) (MedChemExpress HY-12320, USA) for the corresponding time. Western blotting analysis was then performed.

### The Dual-Luciferase reporter assay

Recombinant luciferase reporter plasmids containing the USP11 promoter sequence and Renilla luciferase plasmids were synthesized by the Tsingke Company (Wuhan,China). The experiment was performed in HEK293T cells: the Nrf2-Vector plasmid (2 μg) + recombinant luciferase reporter plasmid containing the USP11 promoter sequence (2 μg) + Renilla luciferase reporter plasmid (0.1 μg); Nrf2 plasmid (2 μg) + recombinant luciferase reporter plasmid containing the USP11 promoter sequence (2 μg) + Renilla luciferase reporter plasmid (0.1 μg). The cells were transfected according to the above groups and treated for the corresponding time, and the dual luciferase reporter assay was performed according to the protocol in the manufacturer.

### Chromatin immunoprecipitation (ChIP)-PCR

Cell sonication was performed using a non-contact ultrasonic disruptor (Biorad). Immunoprecipitation was carried out with Nrf2 antibody and IgG antibody. Semi-quantitative PCR was used to amplify the DNA fragments recovered from immunoprecipitated complexes. The primer sequences are as shown in the Table S3.

### In vivo assays

Male C57BL/6 mice aged 6–8 weeks and male BALB/c nude mice aged 4 weeks were used for the study. The mice were purchased from Beijing Vital River Laboratory Animal Technology. Animal experiments were approved by the Animal Ethics Committee of Wuhan University, with approval number ZN2022042.

### Murine Colitis-associated Cancer (CAC) model

After adapting to the environment for 7 days, the mice were randomly divided into two groups: the Control group and the AOM/DSS group. Mice in the AOM/DSS group were intraperitoneally injected with AOM(10 mg per kg body weight). One week later, we fed the mice with 2% DSS in drinking water for 7 days, followed by normal water for 14 days, and repeated two more cycles. Samples were collected at 10 and 28 weeks after modeling.
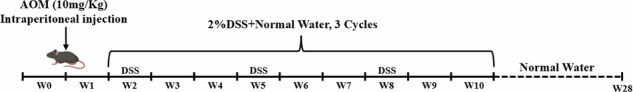


### Xenograft models

We chose the nude mouse subcutaneous transplantation tumor model in vivo experiments, which were randomly divided into three groups: the shNC group, the shUSP11 group, and the shUSP11+TBHQ group, with 5 mice in each group. We injected shNC/shUSPl1 cells (5 × 10^7^ cells) subcutaneously into each mouse (4 weeks, male). When the tumor volume was observed to be stably growing, we administered TBHQ (MedChemExpress HY-100489, USA) to the shUSP11+TBHQ group by intraperitoneal injection, while the other two groups were injected with an equal amount of saline. TBHQ is a widely used Nrf2 agonist with extensive applications in both in vitro and in vivo studies. Based on previous studies, we settled on a dosage of 10 mg/kg [22]. We monitored and recorded the body weight and tumor volume of the nude mice daily. The formula to calculate tumor volume (V) is as follows: V = (L × W^2^)/2, where L represents the longest diameter and W represents the shortest diameter.

### Statistical analysis

The GraphPad Prism software (version 9.0) was employed for data analysis. Results are represented as mean ± SD unless specified stated. Two tailed Student’s *t* test was utilized for comparing two groups, and two-way ANOVA was applied for comparing multiple groups. The significance levels were denoted as follows: *P < 0.05, **P < 0.01, ***P < 0.001, **** *P* < 0.0001, and ns meant no significant statistical difference. All experiments were conducted independently and repeated three times.

## Results

### USP11 was upregulated in CRC tissues

**Fig. 1:**
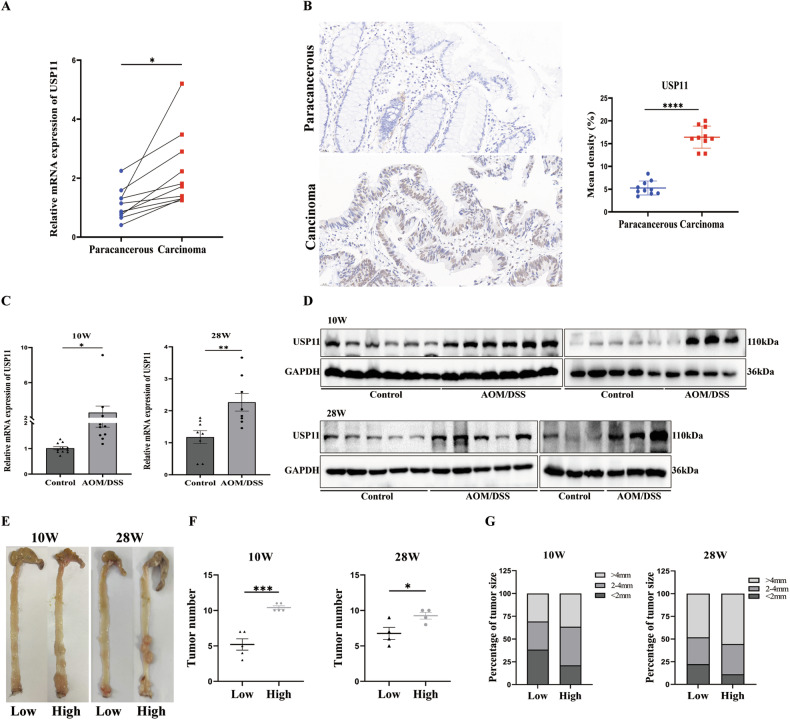
USP11 was upregulated in CRC tissues. **A** Relative expression levels of USP11 mRNA in CRC tissues compared with adjacent normal tissues (*n* = 10) from CRC patients. **B** Representative images of the immunohistochemical staining for USP11 in clinical samples. Scale bar: 20 μm. Statistical analysis of the expression of USP11 in CRC tissues. **C**, **D** Relative mRNA and protein expression levels of USP11 in colorectal homogenates of mice. **E** Macroscopic view of typical colorectal images after mice were euthanized. **F** The number of colorectal tumors in mice was measured. Each dot represents the tumor number of one mouse. **G** The number of tumors of different sizes (diameter) in each mouse were shown.

Our team initiated the investigation by assessing USP11 expression levels in colorectal cancer (CRC) tissues by qPCR and IHC, and found that USP11 was significantly upregulated in cancer tissues (Fig. 1A, B and Fig. S1). Subsequently, we turned to a mouse model of CRC induced by AOM/DSS in order to validate these differences in USP11 expression. The expression level of USP11 in the colon of AOM/DSS mice was significantly higher compared to the normal control group (Fig. 1C, D). Interestingly, this observation was harmonious with the data acquired from clinical CRC specimens, lending weight to the notion that USP11 expression was elevated in CRC, potentially facilitating the progression of the cancer. Based on the expression level of USP11 in the AOM/DSS mouse model, we divided the mice into high- and low- expression groups. In the 10-week and 28-week AOM/DSS mice (Fig. 1E), the number of colon tumors was significantly greater in the high-expression group than in the low-expression group (Fig. 1F). Additionally, based on the size of the mice’s colorectal tumors by the criteria of <2 mm, 2–4 mm and >4 mm in diameter, we observed that tumors were larger in the USP11 high- expression group (Fig. 1G). These findings underscore the likelihood of a role played by USP11 in the advancement of CRC.

### Knockdown of USP11 promoted mitochondrial apoptosis

**Fig. 2:**
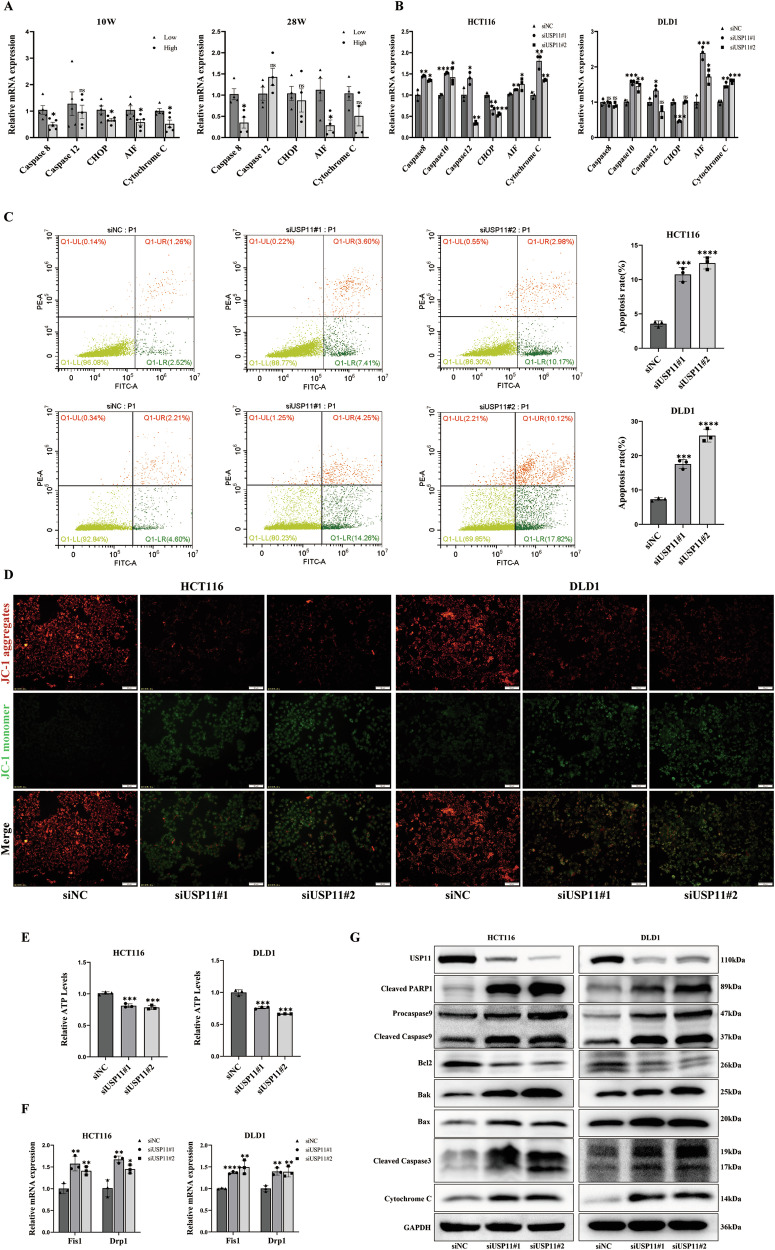
Knockdown of USP11 promoted mitochondrial apoptosis. **A**, **B** The relative mRNA expression levels of apoptosis-related molecules in the USP11 high- and low- expression group in the AOM/DSS mouse model and HCT116/DLD1 cells. HCT116/DLD1 cells were transfected with siRNAs to knock down USP1l. **C** HCT116/DLD1 cells were stained with Annexin V-FITC/ propidium iodide to examine cell apoptosis via flow cytometry. (*n* = 3). **D** Representative fluorescent images of JC-1 monomers and JC-1 aggregates in HCT116/DLD1 cells. (*n* = 3). JC-1 aggregates were stained red, which revealed intact mitochondria. JC-1 monomers, which show the reduction of the ΔΨm, were stained green. Scale bar: 50 μm. **E** Statistical analyses of cellular ATP generation. (*n* = 3). **F** The detection of mitochondrial fission protein. (*n* = 3). **G** Molecules associated with mitochondrial apoptosis: Cleaved PARP1, Procaspase 9, Cleaved Caspase 9, Bcl-2, Bak, Bax, Cleaved Caspase 3, and Cytochrome C levels were measured by western blotting. (*n* = 3).

To further elucidate the effect of USP11 on CRC, we divided AOM/DSS model mice into two groups based on high- and low- expression of USP11, and detected the apoptosis-related markers: extrinsic apoptosis pathway (Caspase8); endoplasmic reticulum stress pathway (Caspase12, CHOP); mitochondrial apoptosis pathway (AIF, Cytochrome C). We found that in the 10-week mouse model, the mRNA levels of Caspase8, CHOP, AIF, and Cytochrome C were lower in the USP11 high-expression group compared to the low-expression group. For the 28-week model mice, the mRNA levels of Caspase8 and AIF were decreased in the high-expression group, suggesting that USP11 promotes the development of CRC by inhibiting its mitochondrial apoptosis (Fig. 2A). Next, we verified whether the differential expression of USP11 was associated with mitochondrial apoptosis in CRC cells. Knockdown of USP11 in HCT116/DLD1 (Fig. S3A) cells resulted in a significant increase in the mRNA levels of extrinsic apoptosis pathway (Caspase8, Caspase10) and mitochondrial apoptosis pathway (AIF, cytochrome C) (Fig. 2B). First, we confirmed that inhibiting the expression of USP11 could promote the apoptosis of CRC cells and inhibited their proliferation (Figs. 2C, Fig. S3B–D). Subsequently, we further explored the effect of USP11 on mitochondrial apoptosis of CRC cells. JC-1 is a fluorescent probe widely used to detect mitochondrial membrane potential. After the inhibition of USP11 in HCT116/DLD1, the red fluorescence intensity decreased significantly, and the green fluorescence intensity increased in HCT116/DLD1 cells (Fig. 2D), and the ATP level decreased (Fig. 2E). Mitochondria, as vital energy-generating organelles, preserved intracellular dynamic balance via ongoing fission and fusion. Disruptions to this balance can alter mitochondrial structure and function, potentially causing mitochondrial fragmentation, network structure loss, and cell apoptosis. Knockdown of USP11 in HCT116/DLD1 cells resulted in increased mRNA levels of mitochondrial fission proteins Fis1 and Drp1 (Fig. 2F), and the expression of mitochondrial apoptotic proteins: Cleaved PARP1, Cleaved Caspase9, Bak, Bax, Cleaved Caspase3, and cytochrome C also increased significantly (Fig. 2G).

### The impact of USP11 on cellular apoptosis was mediated by regulating the Nrf2/ARE signaling pathway

**Fig. 3:**
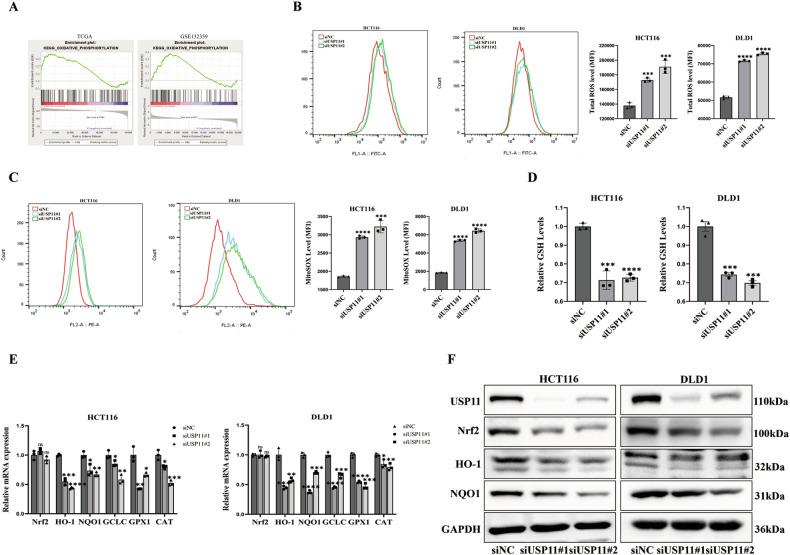
The impact of USP11 on cellular apoptosis was mediated by regulating the Nrf2/ARE signaling pathway. **A** KEGG pathway enrichment analysis. HCT116/DLD1 cells were transfected with siRNAs to knock down USP11 protein. **B**, **C** Flow cytometric detection of total ROS and MitoSOX levels. (*n* = 3). **D** Analysis of GSH levels. (*n* = 3). **E**, **F** qPCR and WB analysis of the expression of Nrf2/ARE, Nrf2 protein level decreased, but its mRNA level was not affected. (*n* = 3).

To further investigate the mechanism by which knocking down USP11 caused mitochondrial apoptosis in CRC cells, we identified the GSE132359 dataset in the GEO database. This dataset involved RNA sequencing of KYSE410 cells, a human esophageal squamous cell carcinoma cell line, in which USP11 was knocked down. Interestingly, our KEGG pathway enrichment analysis illustrated a significant activation of the oxidative phosphorylation pathway following USP11 knockdown. This result was consistent with similar observations from the TCGA dataset (Fig. 3A). Given these insights, we further examined whether USP11 knockdown could induce oxidative stress in CRC cells. Flow cytometry analysis showed that knocking down USP11 in HCT116/DLD1 cells significantly increased total and mitochondrial ROS levels (Fig. 3B, C). What’s more, the measurement of intracellular glutathione (GSH), an important antioxidant, revealed significantly reduced levels in the USP11 knockdown group compared to the NC group (Fig. 3D). These results collectively suggest that USP11 knockdown promoted the production of ROS, thereby inducing oxidative stress in CRC cells. Nrf2 was a widely expressed transcription factor that acted as an antioxidant and could bind to ARE to promote downstream gene expression, enhancing the cell’s antioxidant capacity. The mRNA expression levels of Nrf2 and its downstream antioxidant molecules HO-1, NQO1, GCLC, GPX1, and CAT were significantly lower in the USP11 knockdown group compared to the NC group (Fig. 3E). Western blot analysis also showed a significant decrease in the protein levels of Nrf2 and its downstream HO-1 and NQO1 in the USP11 knockdown group (Fig. 3F), indicating that knocking down USP11 could inhibit the Nrf2/ARE signaling pathway, leading to oxidative stress in cells. Taken together, our findings shed light on the effect of USP11 knockdown in CRC cells, opening avenues for further investigations into its potential as a therapeutic target.

### USP11 protected Nrf2 from degradation via deubiquitination

**Fig. 4:**
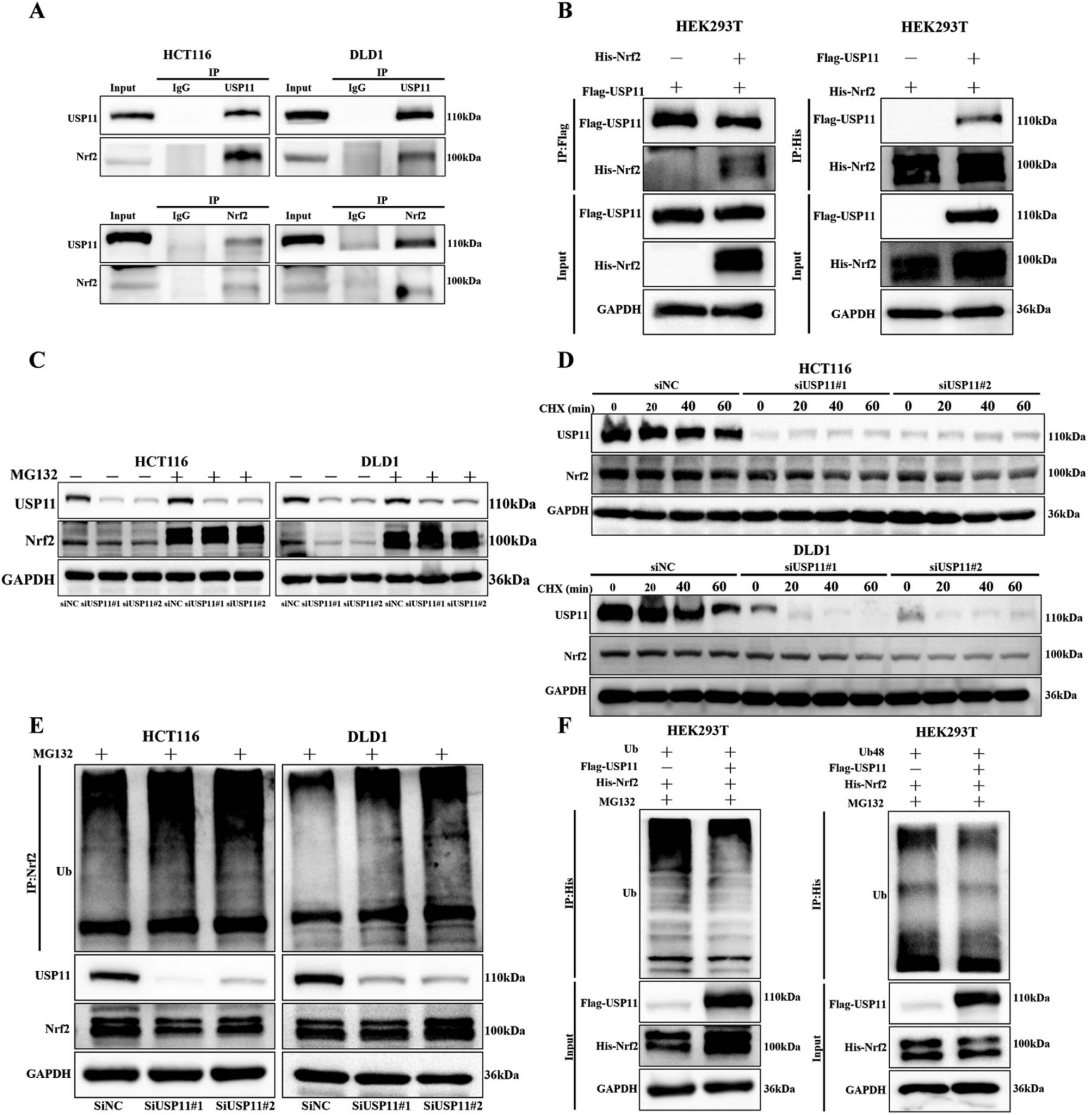
USP11 protected Nrf2 from degradation via deubiquitination. The endogenous interaction of USP11 and Nrf2 was tested in HCT116/DLD1 cell. Flag-USP11, His-Nrf2 plasmids and empty vectors were transfected alone or cotransfected into HEK293 T cells. **A**, **B** USP11/Nrf2 interaction was confirmed by endogenous and exogenous Co-IP assay. (*n* = 3). **C** The decline in Nrf2 protein levels could be reversed by MG132 (20 μM). (*n* = 3). **D** The half-life of Nrf2 was affected by USP11. HCT116/DLD1 cells were treated with CHX (50 μg/ml) and harvested at the indicated times. (*n* = 3). **E** Knockdown of USP11 led to an increase in the polyubiquitination level of Nrf2. (*n* = 3). Ubiquitin, Ubiquitin 48, His-Nrf2 plasmids and Flag-USP11 or empty vector were transfected into 293 T cells and Nrf2 ubiquitination was performed. **F** Functions of USP11 on Nrf2 ubiquitination in HEK293 T cells. USP11 decreased the K48-linked polyubiquitylation of Nrf2. (*n* = 3).

The innate interaction of USP11 and Nrf2 was determined through Co-IP assays in CRC cells in our study (Fig. 4A). Subsequently, we overexpressed Flag-USP11, His-Nrf2, and corresponding control vectors in HEK293T cells to detect their exogenous binding (Fig. 4B). We knocked down USP11 in HCT116/DLD1 cells and treated them with MG132 for 6 h to inhibit the degradation of substrates through the proteasomal pathway. The results showed that MG132 reversed the decrease in Nrf2 protein levels caused by USP11 knockdown (Fig. 4C). We further confirmed the effect of USP11 on the half-life of Nrf2 protein. We knocked down USP11 and used Cycloheximide (CHX) to inhibit the synthesis of new proteins. It was prominently observed that the Nrf2 protein degradation rate was accelerated remarkably within the group of USP11-deficient when compared to the negative control (Fig. 4D). All of the above results confirmed that USP11 regulated Nrf2 through the proteasomal pathway. In HCT116/DLD1 cells, inhibition of USP11 expression resulted in a significant increase in the ubiquitination level of Nrf2 (Fig. 4E). Furthermore, we transfected USP11, Nrf2, and ubiquitin vectors into HEK293T cells, and the results showed that overexpression of USP11 could significantly reduce the ubiquitination level of Nrf2. Additionally, according to the literature, Nrf2 was degraded through K48-linked polyubiquitination. USP11 could also reduce the K48-linked polyubiquitination level of Nrf2 (Fig. 4F).

### USP11 regulated mitochondrial apoptosis in a Nrf2 dependent manner

**Fig. 5:**
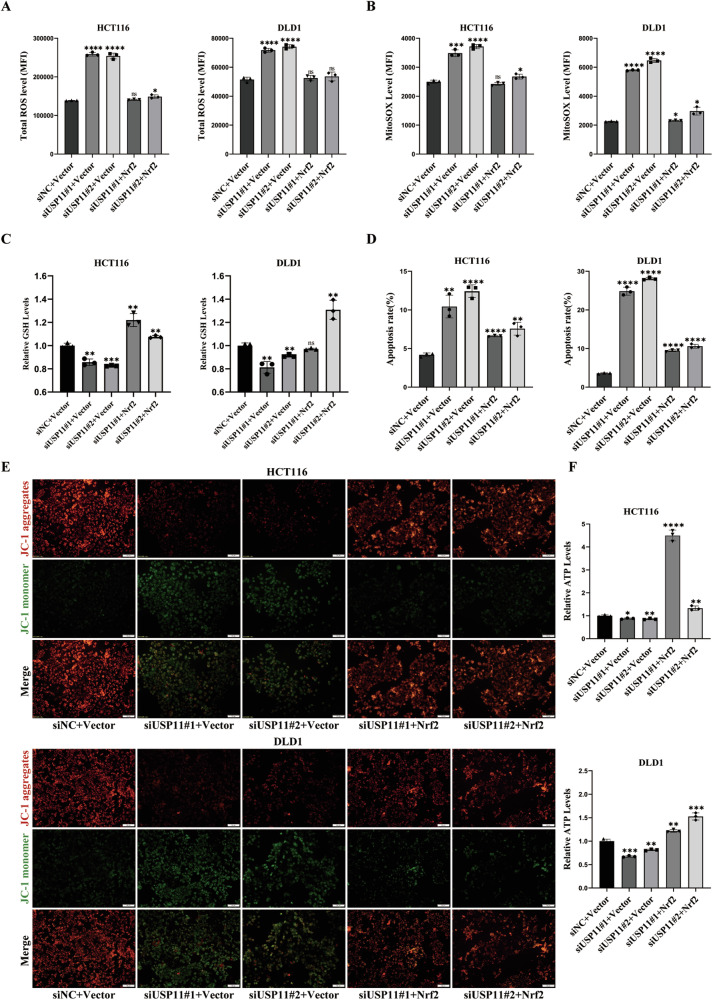
USP11 regulated mitochondrial apoptosis in a Nrf2 dependent manner. HCT116/DLD1 cells were transfected with siRNAs to knock down USP11 protein and then transfected with plasmid to overexpress Nrf2. Statistical analysis of the total ROS (**A**), mitoSOX (**B**), GSH (**C**) and ATP (**F**) levels. (*n* = 3). **D** Apoptosis rate was assayed. Overexpression of Nrf2 could reverse the cell apoptosis caused by USP11 knockdown. (*n* = 3). **E** Representative images by immunofluorescence showed mitochondrial membrane potential. (*n* = 3). Scale bar: 50 μm.

In our studies, we conducted transfection of Nrf2 plasmids following USP11 knockdown. Flow cytometry analysis showed that overexpression of Nrf2 significantly reduced the levels of total ROS and mitochondrial ROS compared to the group with USP11 knockdown (Fig. 5A, B). Subsequently, we detected the intracellular GSH content and discerned a significant surge in its levels upon Nrf2 overexpression (Fig. 5C). We further investigated the effect of overexpressing Nrf2 on mitochondrial apoptosis in CRC cells after knocking down USP11. Overexpression of Nrf2 could significantly reverse the induction of CRC cell apoptosis by USP11 knockdown and promote cell proliferation (Fig. 5D). Activation of Nrf2 in USP11-suppressed group led to a conspicuous upsurge in JC-1 red fluorescence intensity coupled with a noticeable fall in green fluorescence intensity (Fig. 5E). ATP content also increased significantly (Fig. 5F). These results demonstrated that USP11 regulated mitochondrial apoptosis in CRC cells by modulating the Nrf2/ARE signaling pathway.

### USP11 knockdown suppressed CRC proliferation in vivo xenograft model

**Fig. 6:**
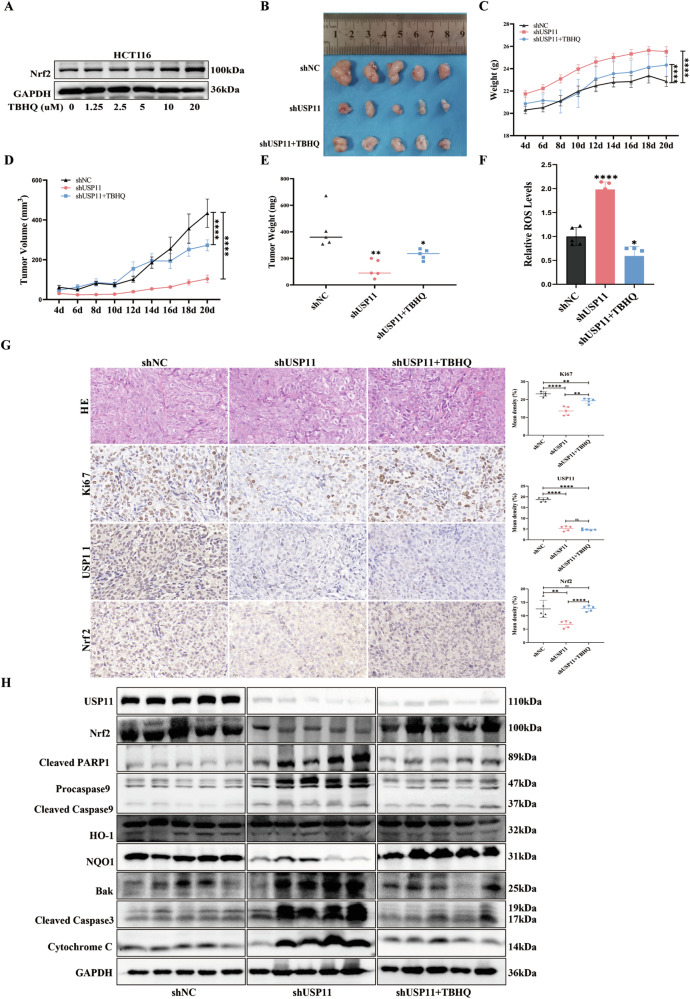
USP11 knockdown suppressed CRC proliferation in vivo xenograft model. **A** TBHQ, a widely used Nrf2 activator, could promote the expression of Nrf2 in HCT116 cell. Nude mice were randomized into three groups and subcutaneously injected with HCT116 cells that had been transfected with control, shUSP11, or shUSP11+TBHQ. **B** Representative photographs of tumors in nude mice were performed. **C** Body weight curves were measured for mice in the three groups. **D**, **E** Statistical analysis graph of the tumor volume and weight. **F** The expression of relative ROS levels, TBHQ could reverse the elevation of ROS caused by the knockdown of USP11. **G** Representative results of IHC staining for Ki67, USP11 and Nrf2. **H** The levels of mitochondrial apoptosis regulatory proteins and Nrf2/ARE signaling pathway protein, including Cleaved PARP1, Procaspase9, Cleaved Caspase9, HO 1, NQO1, Bak, Cleaved Caspase3, Cytochrome C were measured by Western Blotting.

Tert-Butylhydroquinone (TBHQ) was a recognized Nrf2 activator commonly used to promote Nrf2 expression in various cells and animal experiments. First, we confirmed its effect in HCT116 cells (Fig. 6A). In vivo experiments, nude mice were divided into the following three groups: shNC; shUSP11; shUSP11+TBHQ (Fig. S4). We observed a markedly reduced body weight for shNC group as compared to the other two groups (Fig. 6C), but the volume and weight of the subcutaneous tumors were significantly higher than those of the other two groups. Subcutaneous tumors were found to be smallest in terms of volume and lightest in weight in the shUSP11 mice. Interestingly, the activation of Nrf2 significantly promoted subcutaneous tumor growth (Fig. 6B, D, E). Our findings distinctly elucidated that USP11 knockdown could restrictively inhibit the growth of subcutaneous tumors in mice models, yet the inhibitory effect could be reversed by Nrf2. In addition, ROS levels and mitochondrial apoptosis-related protein expression in subcutaneous tumor tissue samples from nude mice with USP11 knockdown were significantly increased, while the expression of antioxidant molecular proteins was significantly decreased. However, Nrf2 activation could significantly reverse the changes in the above indicators (Fig. 6F–H).

### Nrf2 promoted the expression of USP11 at the transcriptional level

**Fig. 7:**
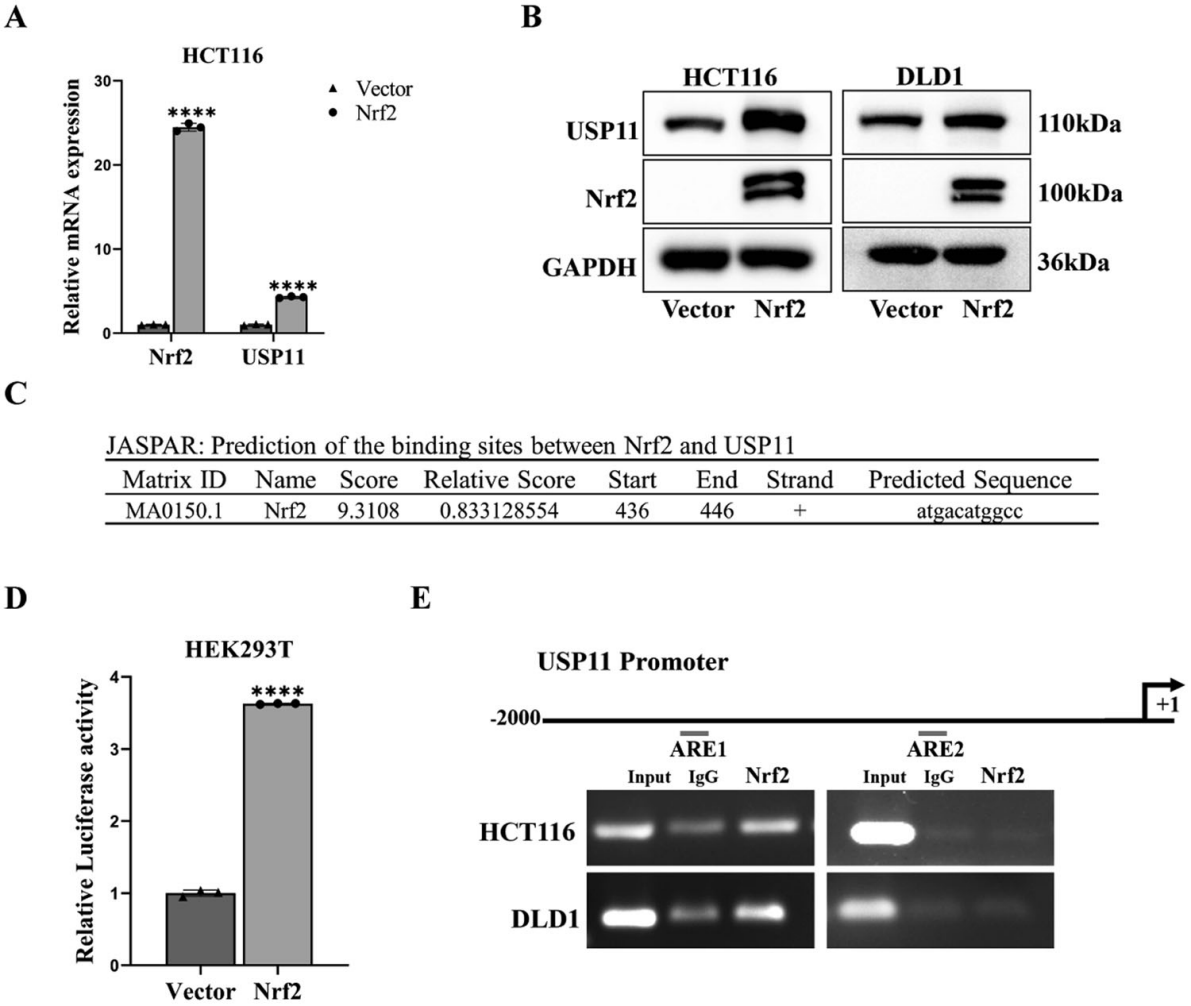
Nrf2 promoted the expression of USP11 at the transcriptional level. **A**, **B** qPCR and WB analysis expression of USP11 following the transfection of Nrf2 plasmid. (*n* = 3). **C** Predicted the binding sites of Nrf2 and the promoter region of USP11 through the JASPAR database. **D** Dual-luciferase reporter assays were performed by co-transfection of Vector or Nrf2 and USP11 promoter reporter constructs. Nrf2 could promote the transcription of USP11. (*n* = 3). **E** Schematic of the locations of the Nrf2 binding site in the USP11 promoter. Nrf2 could bind to the first ARE site in the promoter region of USP11 through the CHIP-PCR assays. (*n* = 3).

Nrf2, was a transcription factor of pivotal significance that promoted the expression of downstream antioxidant genes through its interaction with the ARE sequence. We overexpressed Nrf2 in HCT116/DLD1 cells and detected mRNA and protein levels of USP11 by qPCR and WB (Fig. 7A, B). The results unveiled that Nrf2 could stimulate the expression of USP11. The implications of these findings prompt further investigation into the relationship between Nrf2 and USP11. We predicted that Nrf2 could potentially bind to the tgacatggc base sequence at positions 436-446 of the USP11 promoter by JASPAR software (Fig. 7C). To provide further evidence, we constructed a luciferase reporter plasmid containing the USP11 promoter sequence. In the dual-luciferase reporter assay, we found that the luciferase activity of the Nrf2 group was significantly higher than that of the Vector group, indicating that Nrf2 could bind to the USP11 promoter region (Fig. 7D). Subsequently, we used CHIP experiments to explore the specific binding site of Nrf2 with the USP11 promoter sequence, and the results showed that Nrf2 promoted its transcription by binding to the ARE1 sequence of the USP11 promoter (Fig. 7E).

## Discussion

The occurrence of CRC was a multifaceted process influenced by a variety of factors such as genetics and environmental conditions. Currently, the treatment efficacy for most CRC patients was unsatisfactory. It has been reported that USP11 was highly expressed in various cancerous tissues such as lung cancer, breast cancer, and ovarian cancer, and promoted the progression of tumors [15, 23–25]. The role of USP11 varied as it controled different substrate proteins [26–29]. In CRC, USP11 could promote drug resistance, proliferation, and invasion, However, it was yet to be ascertained whether it had a direct effect on inducing apoptosis in CRC cells. Thus, it called for additional research to be conducted on this topic. This study would be devoted to examining the effect of USP11 on the apoptosis of CRC cells and the related molecular mechanisms.

Evidence strongly suggests that USP11 promotes the proliferation and migration of CRC by maintaining stability of PPP1CA or IGF2BP3 through deubiquitination [30, 31]. Recently, a new study revealed that Cicr-DOCK1 interacts with miR-132-3p to inhibit its effect on USP11, thereby promoting the proliferation, invasion, and migration of CRC [32]. Moreover, USP11 has been cast under the spotlight for its association with drug resistance in CRC [33, 34]. In this study, we first found that USP11 was highly expressed in the AOM/DSS mouse model. The pertinence of apoptosis as a dominant mechanism of cellular demise within tumor cells, inclusive of extrinsic and intrinsic pathways, is undeniable. The latter can be further branched into two paths: endoplasmic reticulum stress and mitochondrial apoptosis pathway. Based on the expression level of USP11 in the AOM/DSS mouse model, we divided the mice into high- and low- USP11 expression groups, and then detected apoptosis-related indicators in these two groups. Our observations highlighted that the levels of Caspase8 and AIF mRNA in the USP11 high-expression group were lower than those in the USP11 low-expression group, indicating that USP11 might play a role by inhibiting CRC mitochondrial apoptosis. Next, in vitro experiments, we knocked down USP11 in HCT116/DLD1 cells and detected the relevant indicators of mitochondrial apoptosis: mitochondrial membrane potential, ATP content, and proteins associated with mitochondrial apoptosis. We clearly found that knocking down USP11 could promote CRC cell mitochondrial apoptosis.

Using data from GSE132359 and TCGA, we conducted KEGG pathway enrichment analysis and found that the oxidative phosphorylation pathway was significantly enriched in the USP11 low-expression group. Nrf2 (NFE2L2), a key intracellular antioxidant molecule, had seven Neh (Nrf2-ECH homology) domains, namely Neh1-Neh7. The CNC-bZIP region in Neh1 could form heterodimers with sMaf proteins, recognized and bound to ARE, promoting downstream expression of antioxidant genes [5], which was crucial for maintaining intracellular homeostasis. Mitochondria were both the source and target of intracellular ROS, meanwhile, ROS-induced oxidative stress damage could lead to mitochondrial dysfunction and cell death [35–37]. Therefore, Nrf2 and mitochondrial apoptosis had grabbed significant attention. After knocking down USP11, we detected the expression levels of Nrf2 and its downstream antioxidant molecules and found that the mRNA levels of NQO1, HO-1, GCLC, GPX1, and CAT were significantly reduced. Concurrently, the protein levels of Nrf2, HO-1, and NQO1 were also significantly decreased. These findings suggested that USP11 could regulate the Nrf2/ARE signaling pathway, leading to the oxidative stress in cells. In addition, USP11 could affect the protein level of Nrf2 but not its mRNA level. Therefore, we speculated that the deubiquitinating enzyme USP11 inhibited the ubiquitin-proteasome pathway degradation of Nrf2, which was confirmed in subsequent experiments.

There were few reports on the upstream molecules of USP11. In hepatocellular carcinoma research, it had been observed that USP11 might stabilize the protein level of the transcription factor E2F1, and E2F1 could also stimulate the transcription and translation of USP11 [16]. The positive feedback loop between the two might promote the progression of hepatocellular carcinoma. Nrf2, as a transcription factor, could bind to the ARE sequence in the downstream gene promoter region, thus advancing the downstream genes’ expression. Nrf2 had been reported to form feedback regulation mechanisms with many molecules, such as Gankyrin, TIGAR, FGF21, SIRT1, HBXIP, and P62, thereby affecting the development of diseases [38–43]. We hypothesized that Nrf2 might also affect the expression of USP11, potentially forming a feedback regulation loop, which might impact the development of CRC. To verify this hypothesis, we first overexpressed Nrf2 and confirmed that Nrf2 could promote the expression of USP11. Afterwards, we searched for the sequence of the human USP11 promoter region and discovered two ARE sequences. Following dual luciferase reporter gene experiments and CHIP-PCR results, it was established that Nrf2 bound to the first ARE sequence in the USP11 promoter region, thereby promoting the transcription of USP11.

## Conclusion

To sum up, our study found that USP11 was highly expressed in CRC tissues and was associated with poor prognosis. Inhibiting USP11 expression in CRC cells could promote the ubiquitination and degradation of Nrf2, thereby inhibiting NRF2/ARE signaling pathway, increasing intracellular ROS, and leading to mitochondrial apoptosis in CRC cells. In addition, Nrf2 could bind to the USP11 promoter region to enhance its transcription, forming a positive feedback loop.

## Supplementary information

Supplementary data

Original Data

## Data Availability

The novel contributions made in this study can be found within the article and the provided Supplementary Material. The gene expression data were procured from the GEO database (https://www.ncbi.nlm.nih.gov/geo/) and the TCGA database (https://portal.gdc.cancer.gov/). For any further inquiries, please refer to the corresponding authors.
