# Supplementary material for: The USP11/Nrf2 positive feedback loop promotes colorectal cancer progression by inhibiting mitochondrial apoptosis
Source: Cell Death Dis. 2024 Dec 1;15(12):873. doi: 10.1038/s41419-024-07188-2 (PMC11609304; doi:10.1038/s41419-024-07188-2)
Supplement: Supplementary file 1 — Supplementary data [file 41419_2024_7188_MOESM1_ESM.pdf]

Supplementary Information

Summary

Figure S1-4 and the corresponding figure legends.

Table S1: Sequences of primers for PCR.

Table S2: Sequences of siRNA.

Table S3: Sequences of primers for CHIP-PCR.

Supplementary Figures and Figure legends

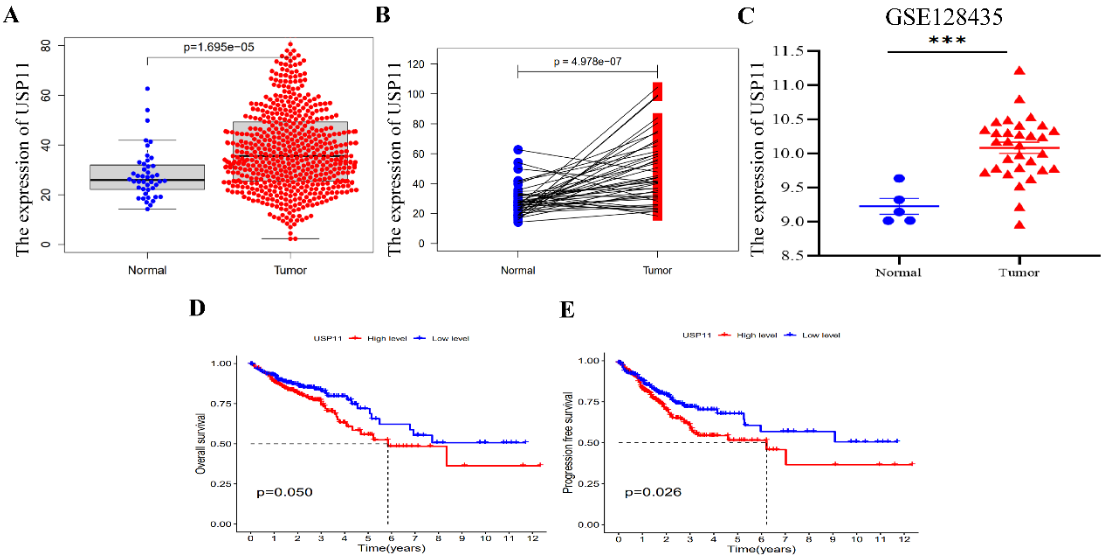

Figure S1 USP11 was highly expressed in CRC tissues and was associated with a poor prognosis. Analysis of the TCGA database (A) and GSE128435 (C) showed that USP11 was highly expressed in CRC tissues. (B) USP11 expression in the paired CRC tissues and adjacent normal tissues. (D-E) Kaplan–Meier survival analysis evaluating the prognosis and progression free survival (PFS) in patients expressing high or low expression of USP11.

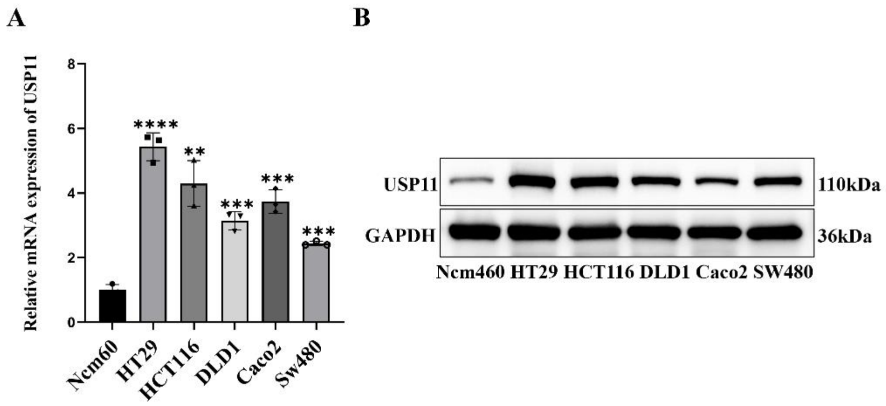

Figure S2 USP11 was overexpressed in CRC cells. (A-B) Western blotting analysis and qPCR of USP11 expression in the normal human colon cell line and CRC cell lines. (n=3).

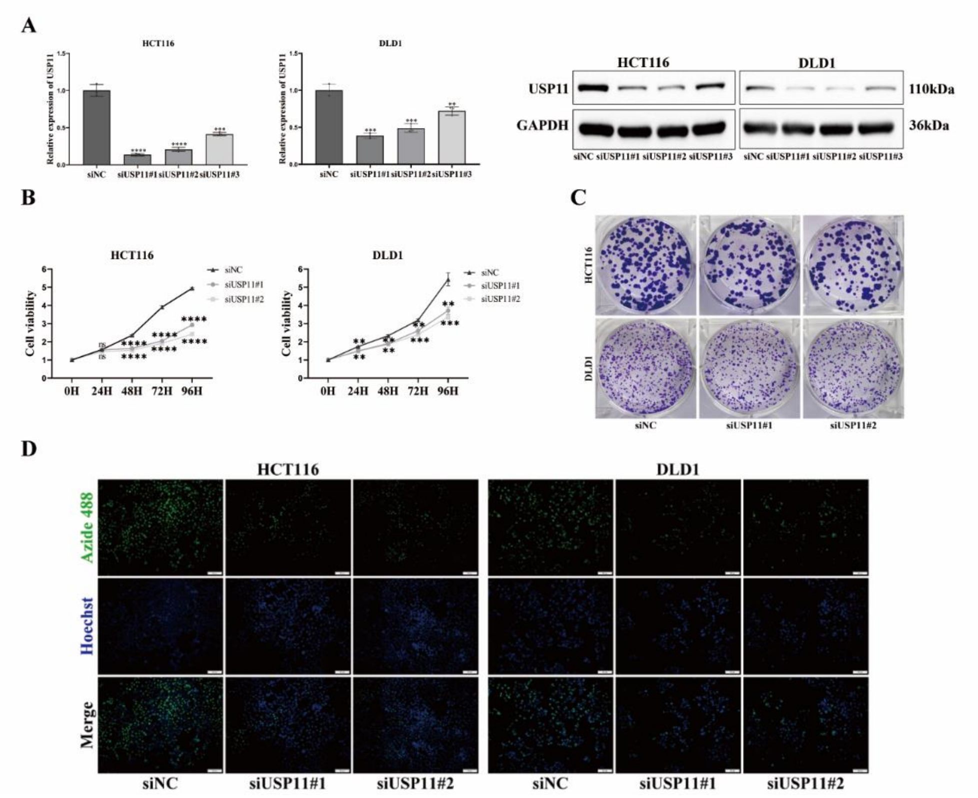

Figure S3 Suppression of USP11 inhibited CRC cell proliferation in vitro. (A) USP11 knockdown efficiency were confirmed by qPCR and western blotting. The proliferation of HCT116/DLD1 cells were examined by CCK-8 assay(B), colony formation assay (C) and EdU assay (D) after USP11 knockdown. (n=3). EdU positive (Alexa Fluor 488 azide labeled; green) and 1× Hoechst stained nuclei of all the cells (blue) were visualized by fluorescence microscopy.

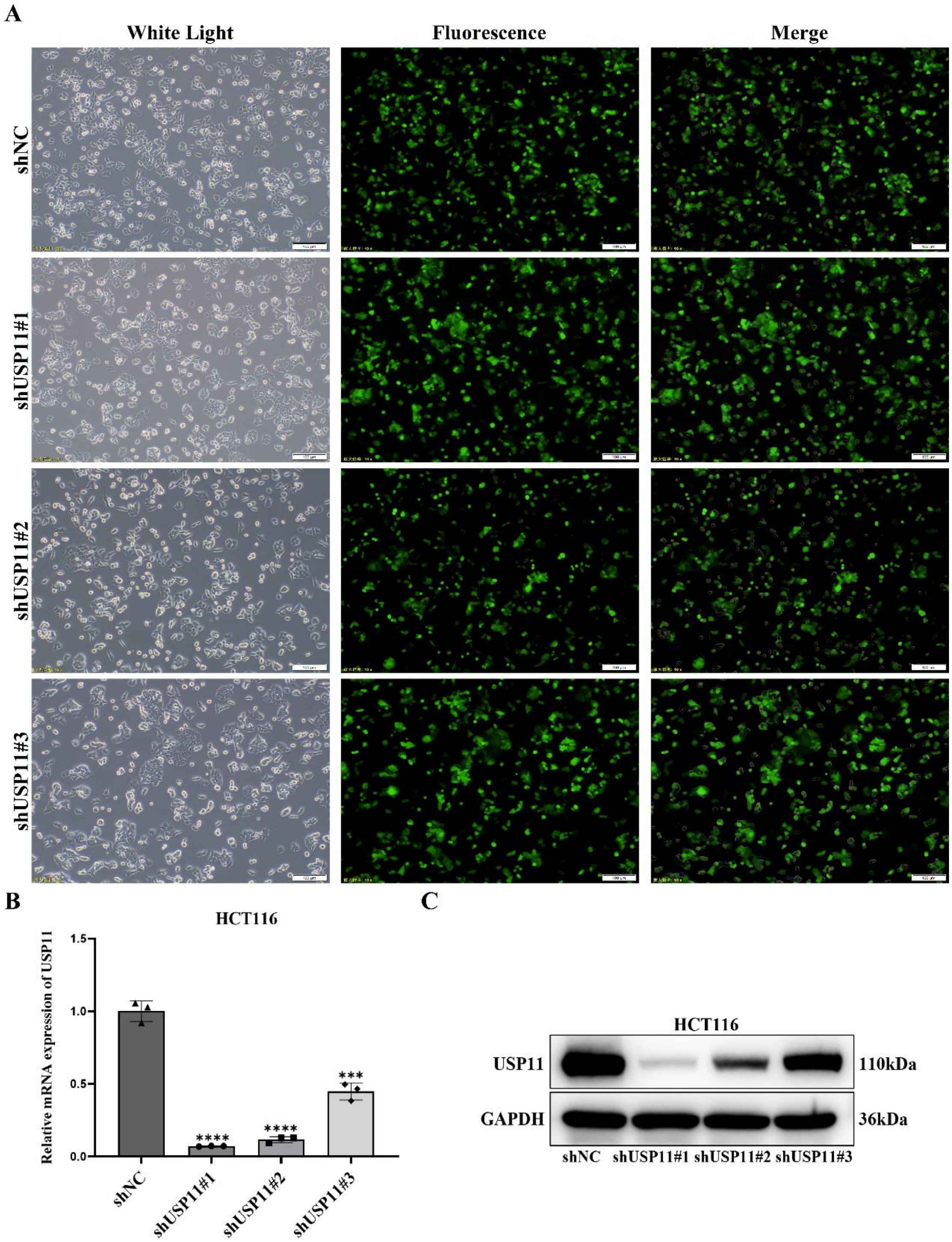

Figure S4 Lentivirus-mediated silencing of USP11 in HCT116 cell. (A) Fluorescence micrographs of HCT116 cells infected with lentivirus at a magnification of 100×. (n=3). (B-C): Validation of the USP11 knockdown efficiency in HCT116 cells. GAPDH protein was used as control. (n=3).

Table S1. Sequences of primers for PCR.

Gene		PCR
Hum-USP11	5'Primer	GAGGATGATGGTGGCTGATGTCTTC
	3'Primer	CACGGTCCAAGATGCTGCTCAG
Hum-Caspase8	5'Primer	CAGAGCCTGAGAGAGCGATG
	3'Primer	AGGCTGAGGCATCTGTTTCC
Hum-Caspase10	5'Primer	GACAAGGAAGCCGAGTCGTA
	3'Primer	ATGACTCAGGATCTCATTACTACCT
Hum-Caspase12	5'Primer	TTGACCTTTTGGGGATGCGA
	3'Primer	CAGTGCTTGGTCCCACAGAT
Hum-CHOP	5'Primer	TCCAACTGCAGAGATGGCAG
	3'Primer	CAGTCAGCCAAGCCAGAGAA
Hum-AIF	5'Primer	ATCCGTTGGAGTCAGCAGTG
	3'Primer	ACCCAGATGTTAGAGCGTGC
Hum-Cytochrome C	5'Primer	GCCACACCGTTGAAAAGGGA
	3'Primer	TTGGCGGCTGTGTAAGAGTAT
Hum-Nrf2	5'Primer	AGTCCAGAAGCCAAACTGACAGAAG
	3'Primer	GGAGAGGATGCTGCTGAAGGAATC
Hum-HO-1	5'Primer	CAAGGAGAGCCCAGTCTTCG
	3'Primer	CTTCACATAGCGCTGCATGG
Hum-NQO1	5'Primer	GAAAGGCTGGTTTGAGCGAG
	3'Primer	GCAGAGAGTACATGGAGCCAC
Hum-Fis1	5'Primer	AGGCCTTAAAGTACGTCCGC
	3'Primer	ACAGCAAGTCCGATGAGTCC
Hum-Drp1	5'Primer	GGTCATGCCGTGAACCTGCT
	3'Primer	TTCTTTCCTGCGCTGTGCCA
Hum-GCLC	5'Primer	AGAGAAGGGGGAAAGGACAAAC
	3'Primer	AAGTTATTGTGCAAAGAGCCTGAT
Hum-GPX1	5'Primer	TGCAACCAGTTTGGGCATCA
	3'Primer	ACCGTTCACCTCGCACTTC
Hum-CAT	5'Primer	ACTTTGAGGTCACACATGACATT
	3'Primer	CTGAACCCGATTCTCCAGCA
Hum-GAPDH	5'Primer	GTCTCCTCTGACTTCAACAGCG
	3'Primer	ACCACCCTGTTGCTGTAGCCAA
Mus-USP11	5'Primer	TTGATGCTGTCCTGAGTGGGTTTG
	3'Primer	GCACTTAGGAGGCAGAGGTACAATC
Mus-Caspase8	5'Primer	TGAGGCAGACTTTCTGCTGG
	3'Primer	CTCAGGCTCTGGCAAAGTGA
Mus-Caspase12	5'Primer	GCGGCCAGGAGGACACAT
	3'Primer	AAACCCCATCCAGCATGTCC

Mus-CHOP	5’Primer	AACAGAGGTCACACGCACAT
	3’Primer	ACTTTCCGCTCGTTCTCCTG
Mus-AIF	5’Primer	CAGAAACTGGTGCCCTTGGT
	3’Primer	GGAACAAGTTGCCTGGAAGC
Mus-Cytochrome C	5’Primer	GACCAAATCTCCACGGTCTGT
	3’Primer	GGTATCCTCTCCCCAGGTGAT
Mus-GAPDH	5’Primer	CATCACTGCCACCCAGAAGACTG
	3’Primer	ATGCCAGTGAGCTTCCCGTTCAG

Table S2. Sequences of siRNA

siRNA		Sequences
hsa-USP11	#1	GCCCGTGACTACAACAACT
	#2	GGTCGAAGTGTACCCAGTA
	#3	AGCCGTACATTGCTATCGA

Table S3. Sequences of primers for CHIP-PCR.

Binding site		PCR
ARE1	5’Primer	GTCCCCAGAGATGGAAAAGCA
	3’Primer	GGCAATCCTGTCCCCCTTTG
ARE2	5’Primer	GAAATGACAGTGCTGAGGTTGAG
	3’Primer	AGGAATCCCGAGACCCAAAGTT
