# Supplementary material for: The USP11/Nrf2 positive feedback loop promotes colorectal cancer progression by inhibiting mitochondrial apoptosis
Source: Cell Death Dis. 2024 Dec 1;15(12):873. doi: 10.1038/s41419-024-07188-2 (PMC11609304; doi:10.1038/s41419-024-07188-2)

Figure1D


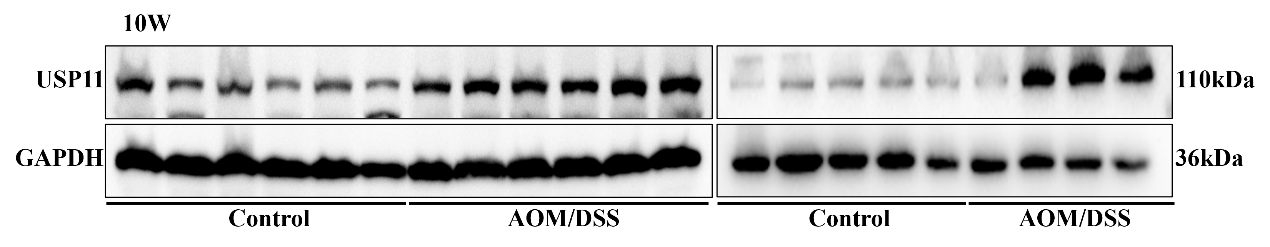


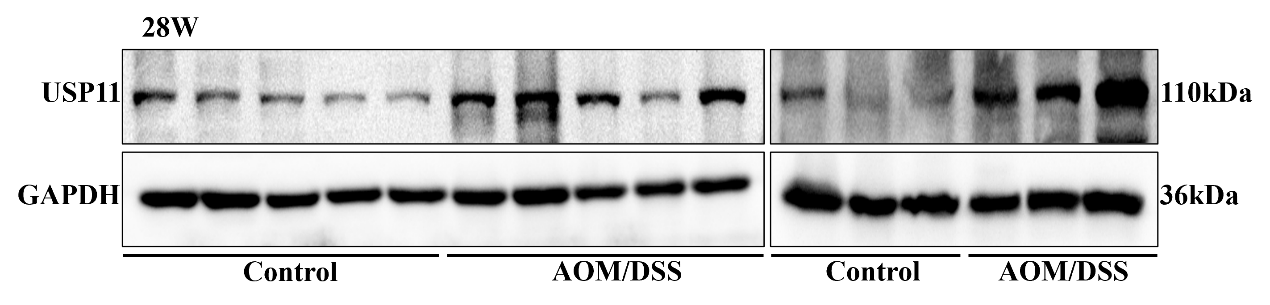


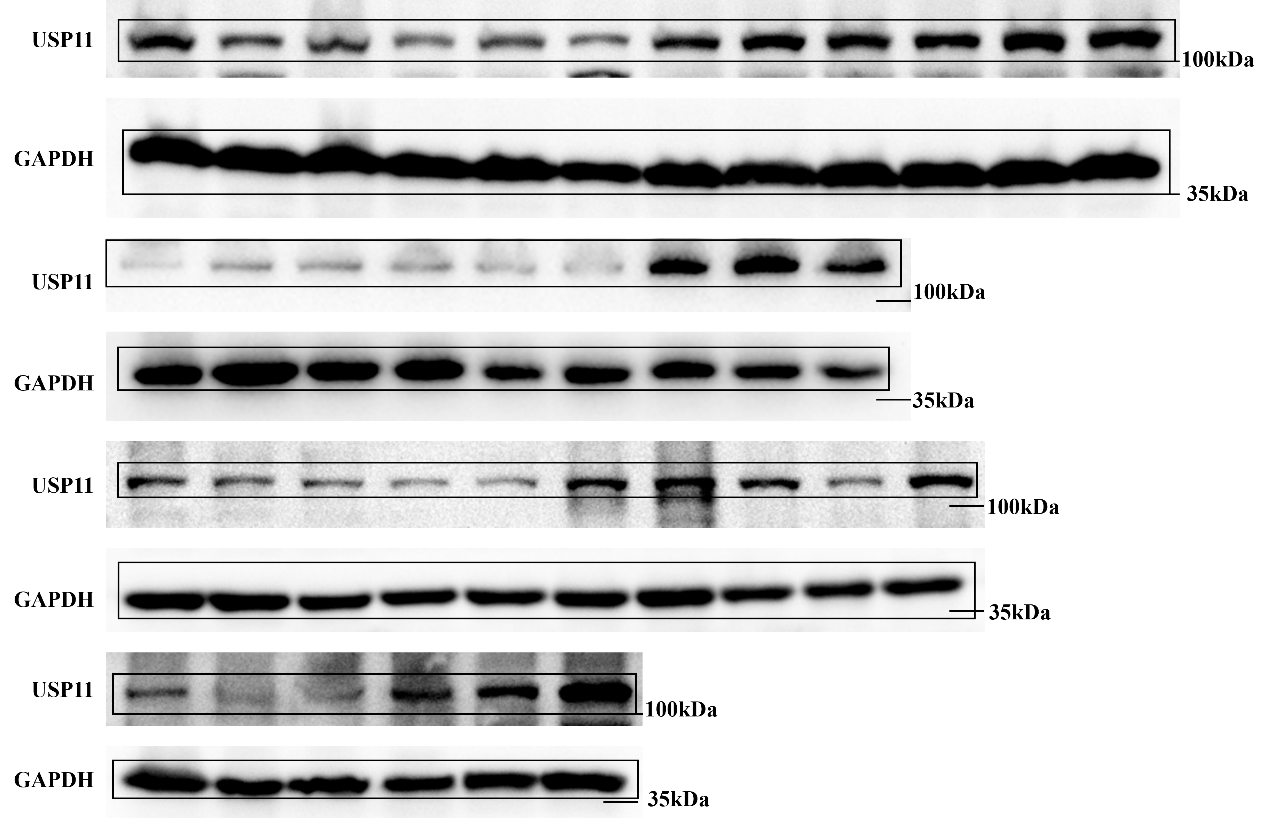


Figure2G


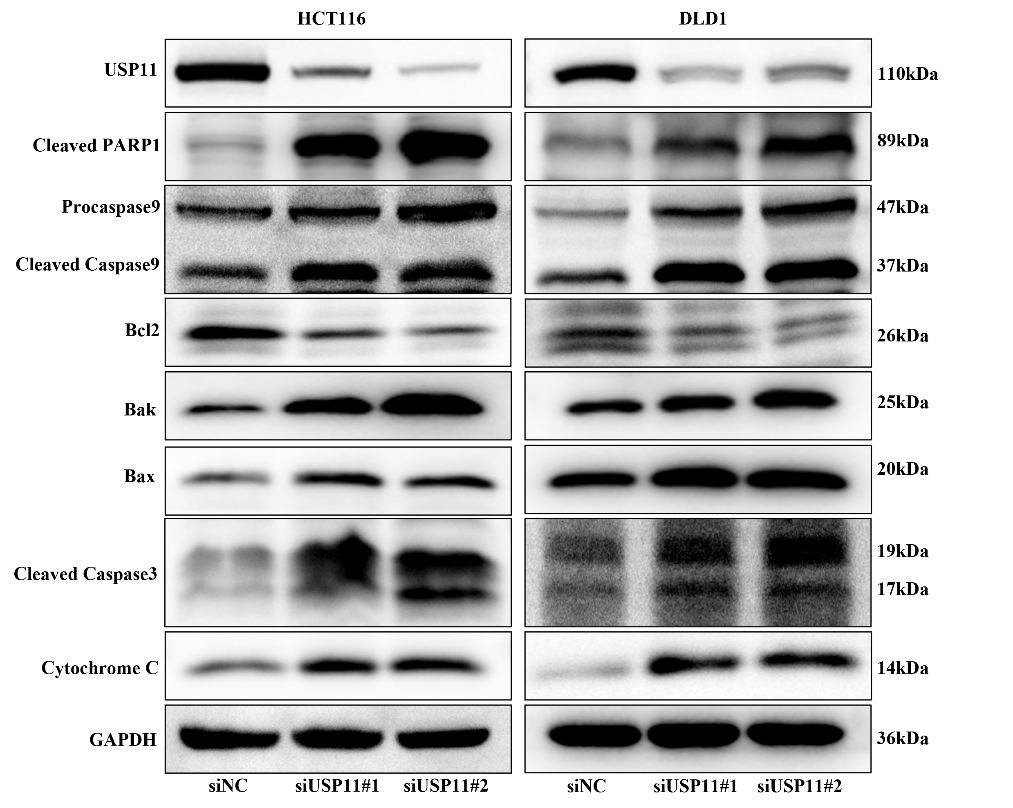


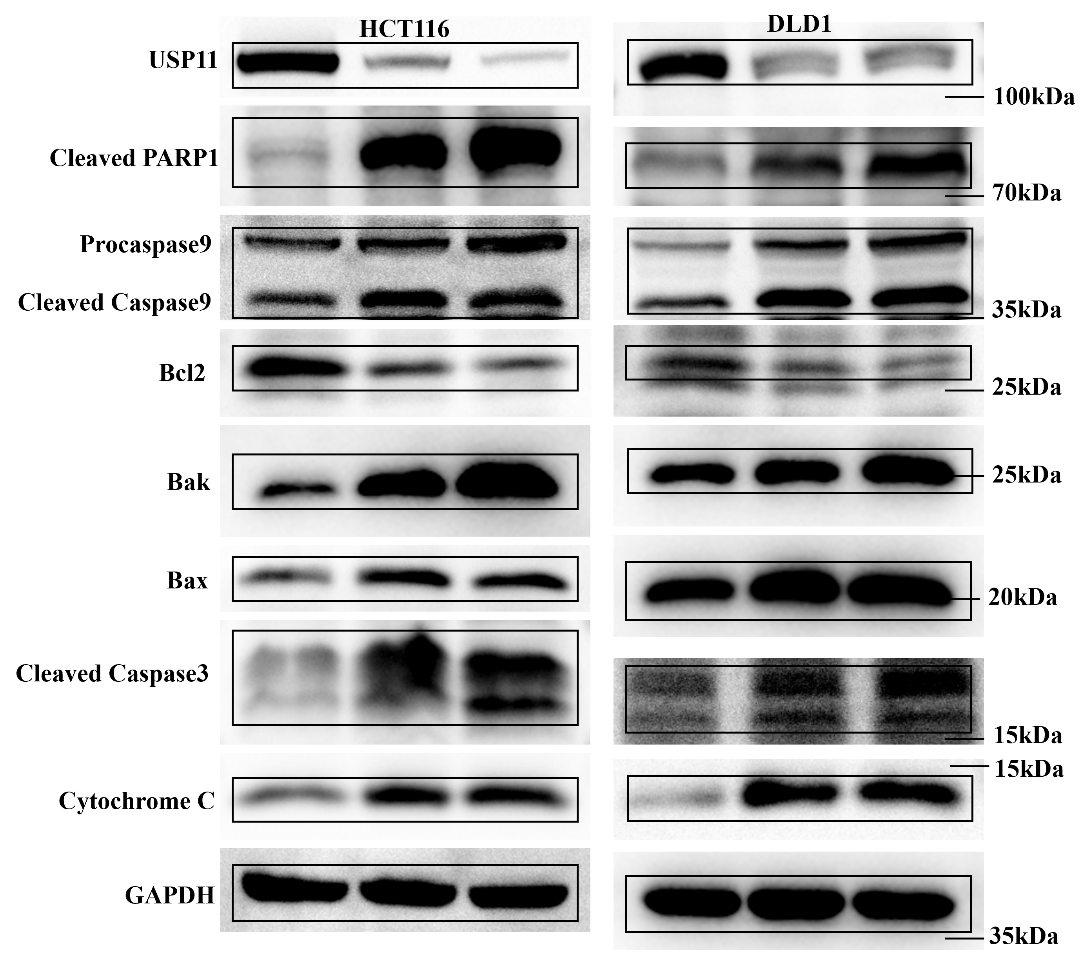


Figure3F


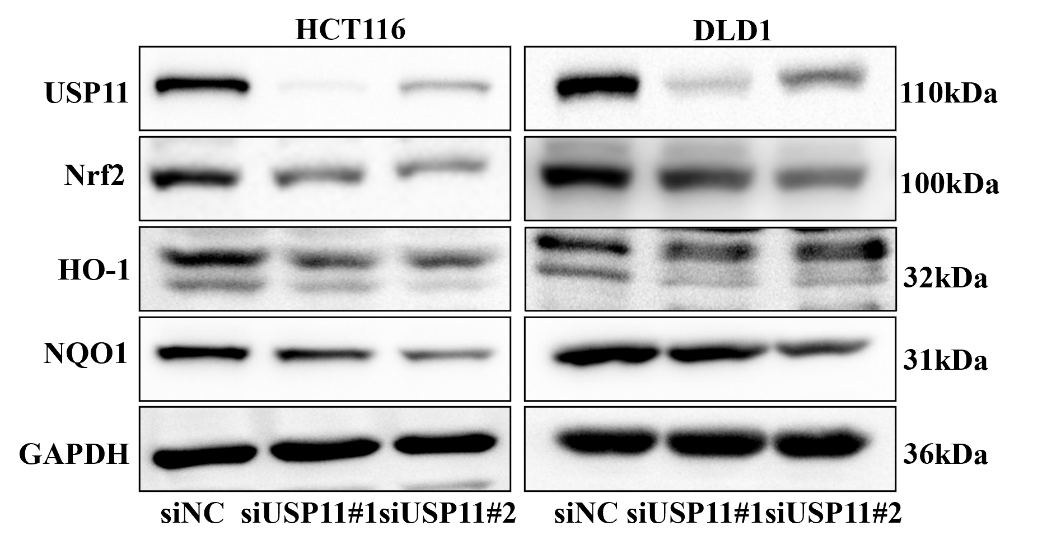


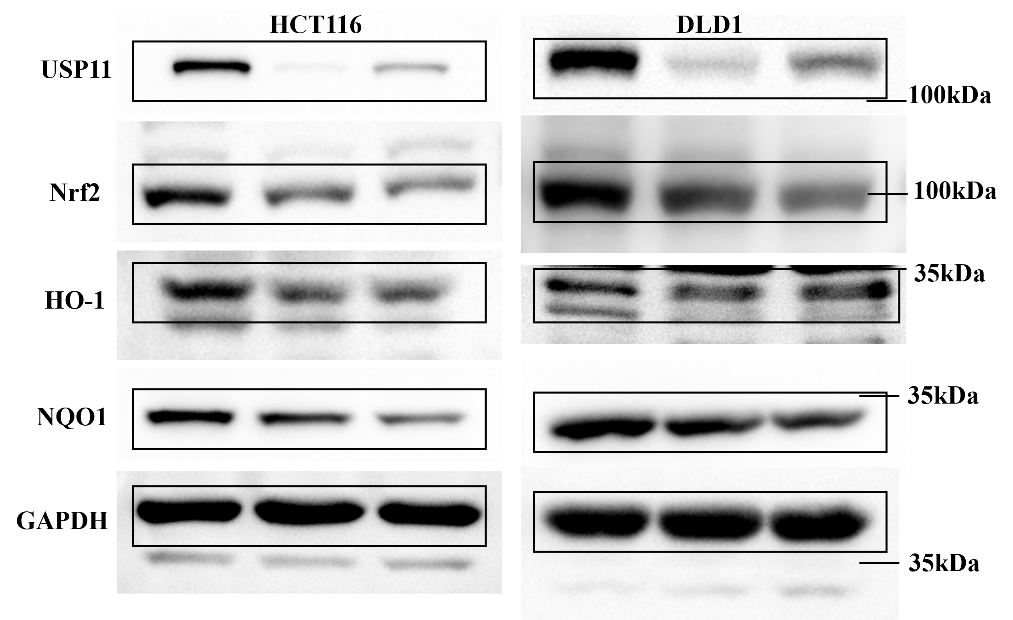


Figure4A


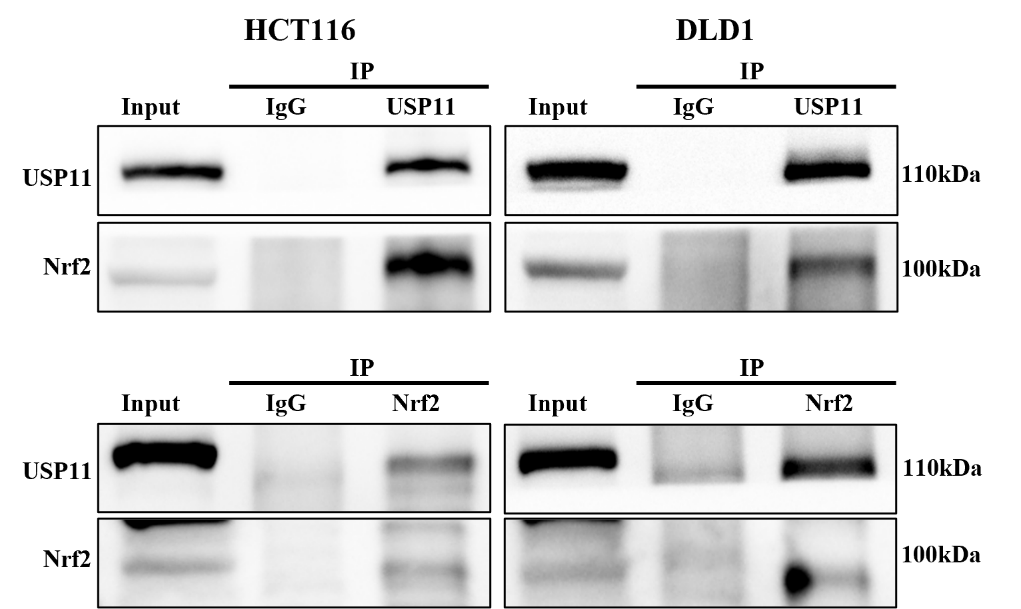


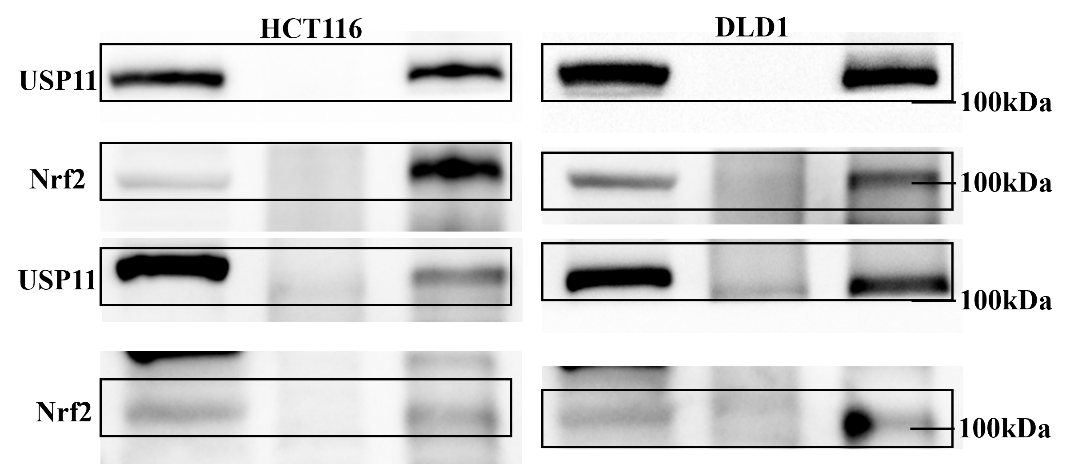


Figure4B


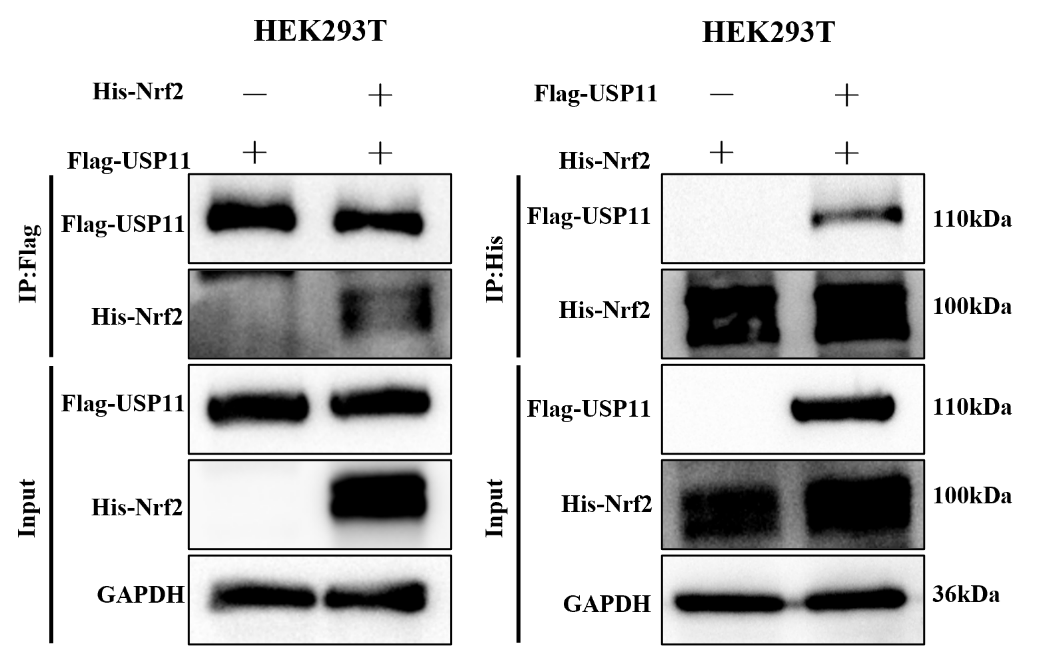


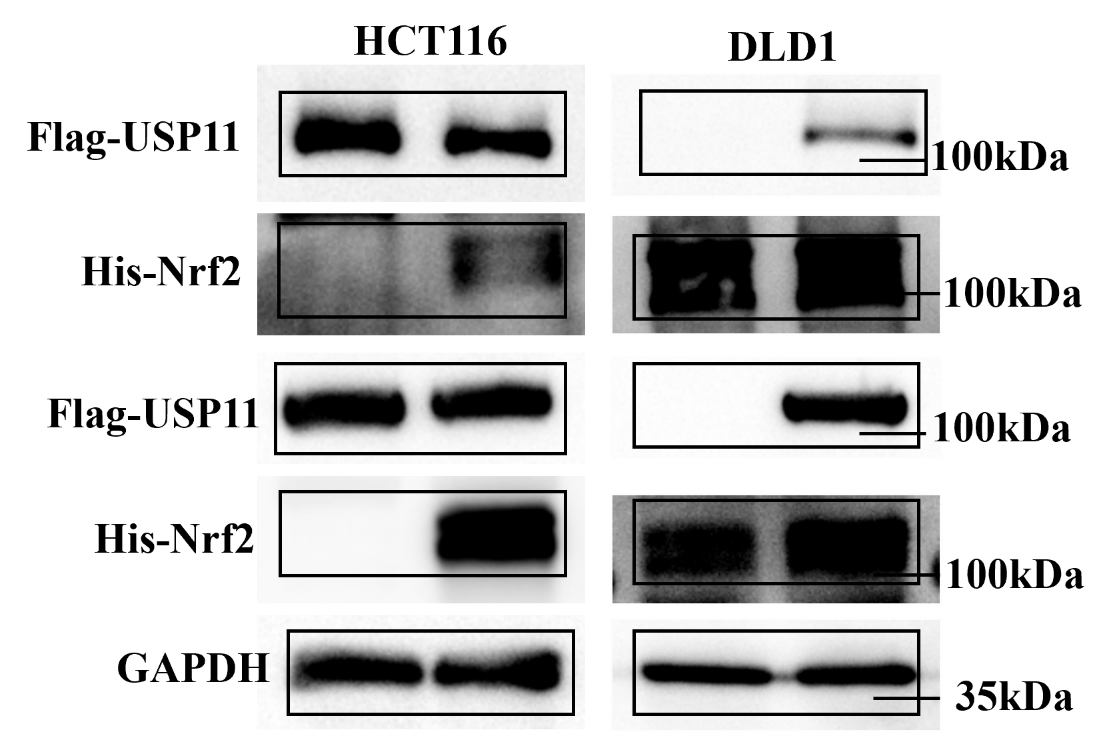


Figure4C


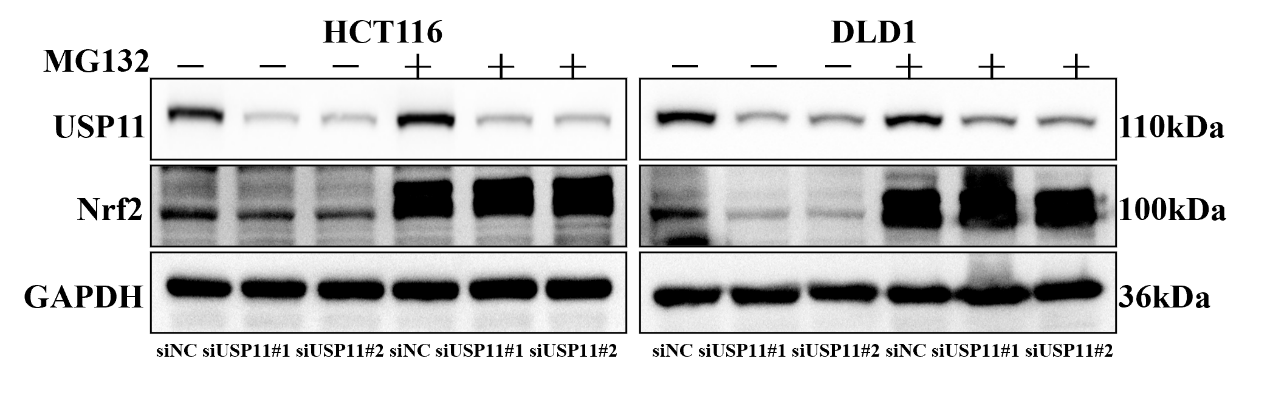


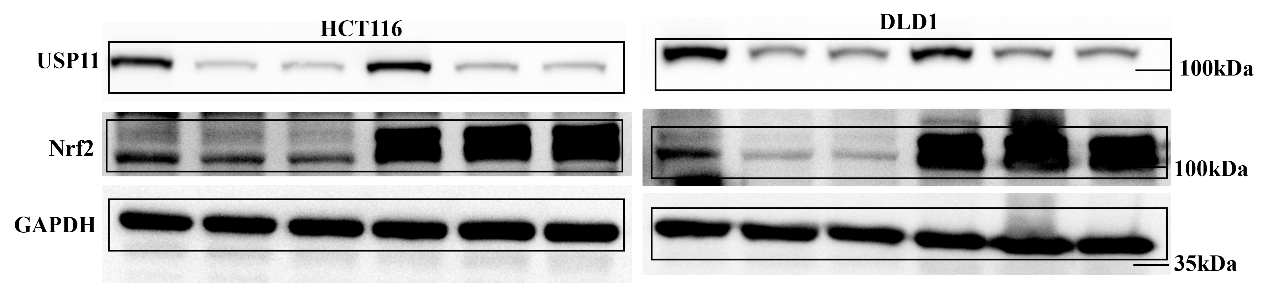


Figure4D


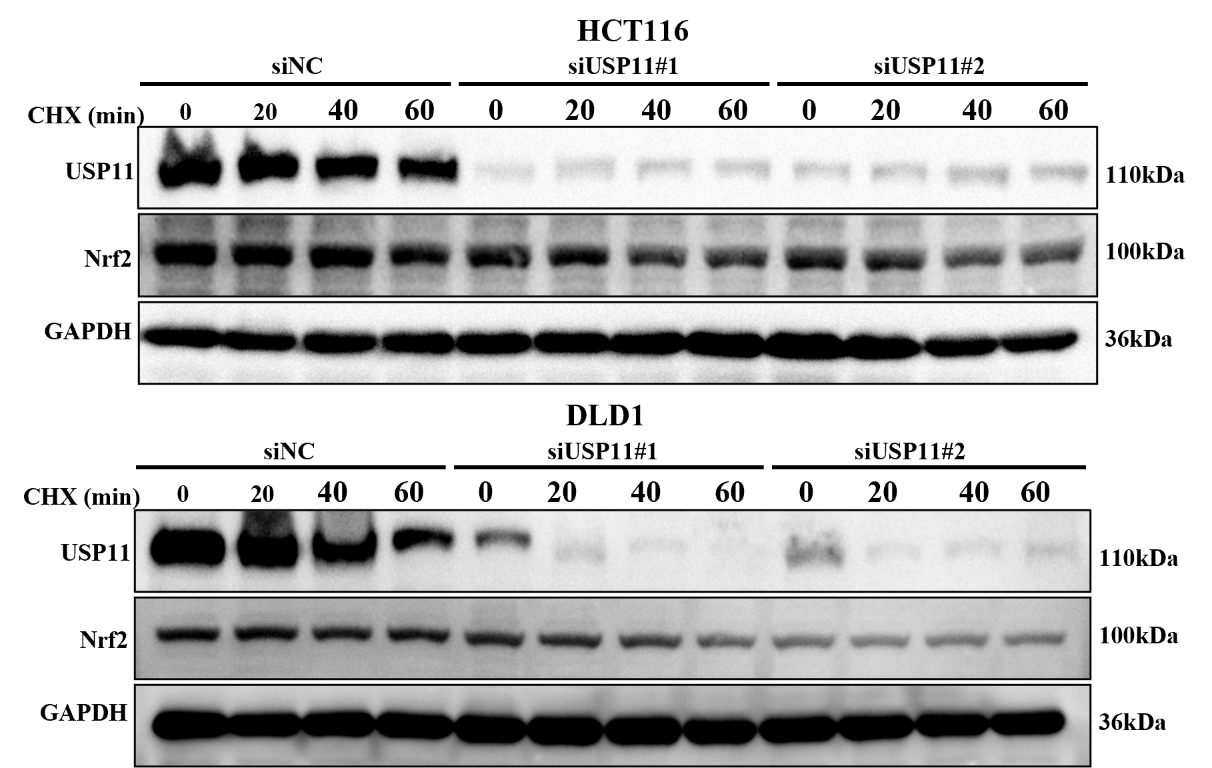


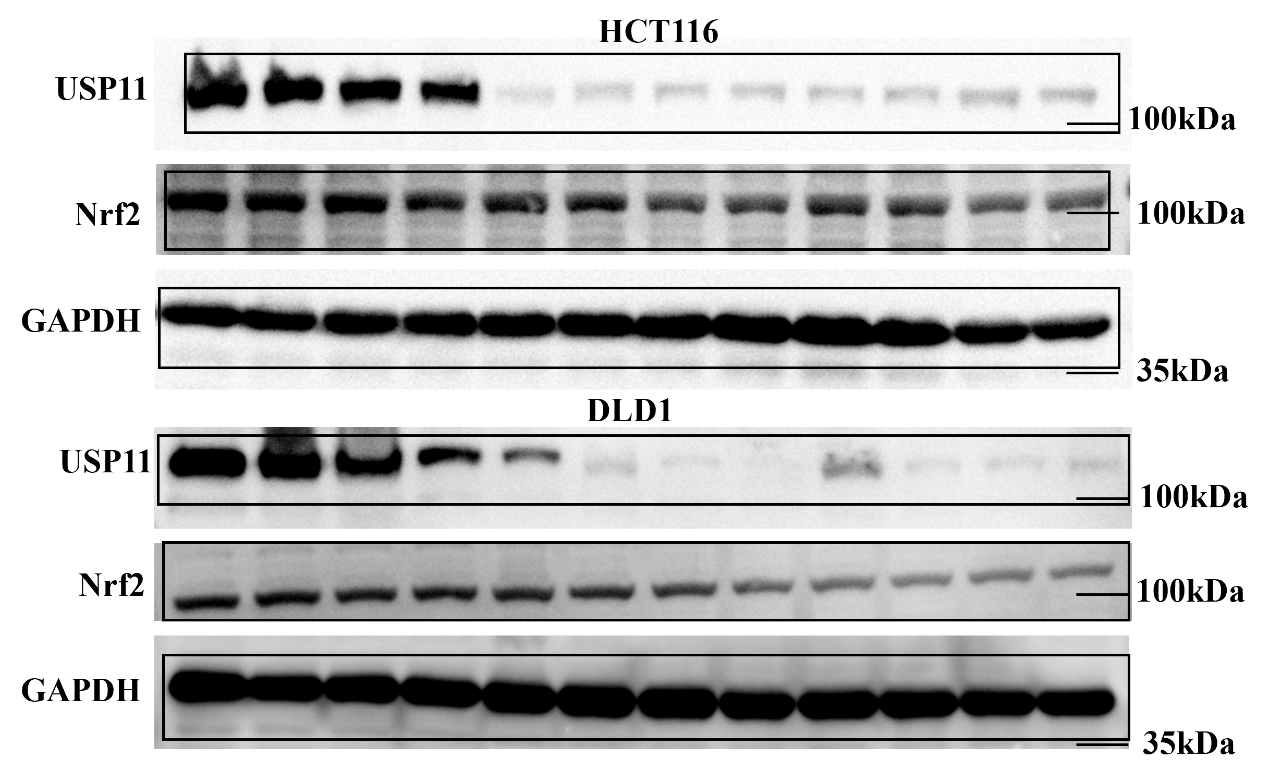


Figure4E


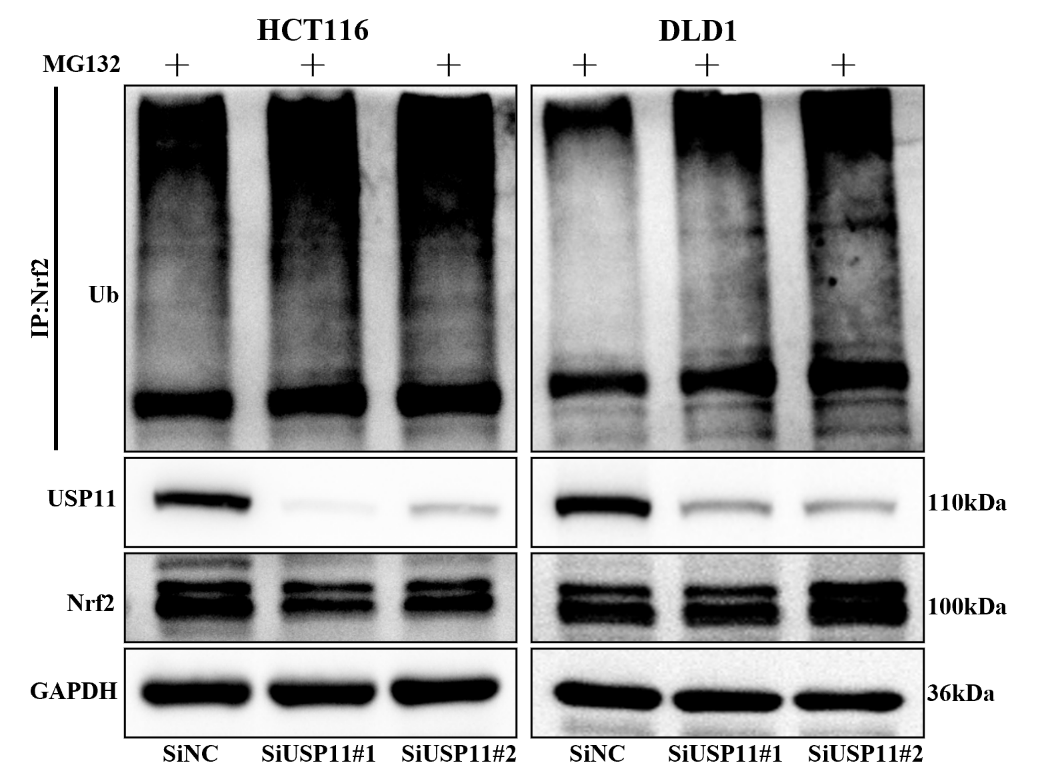


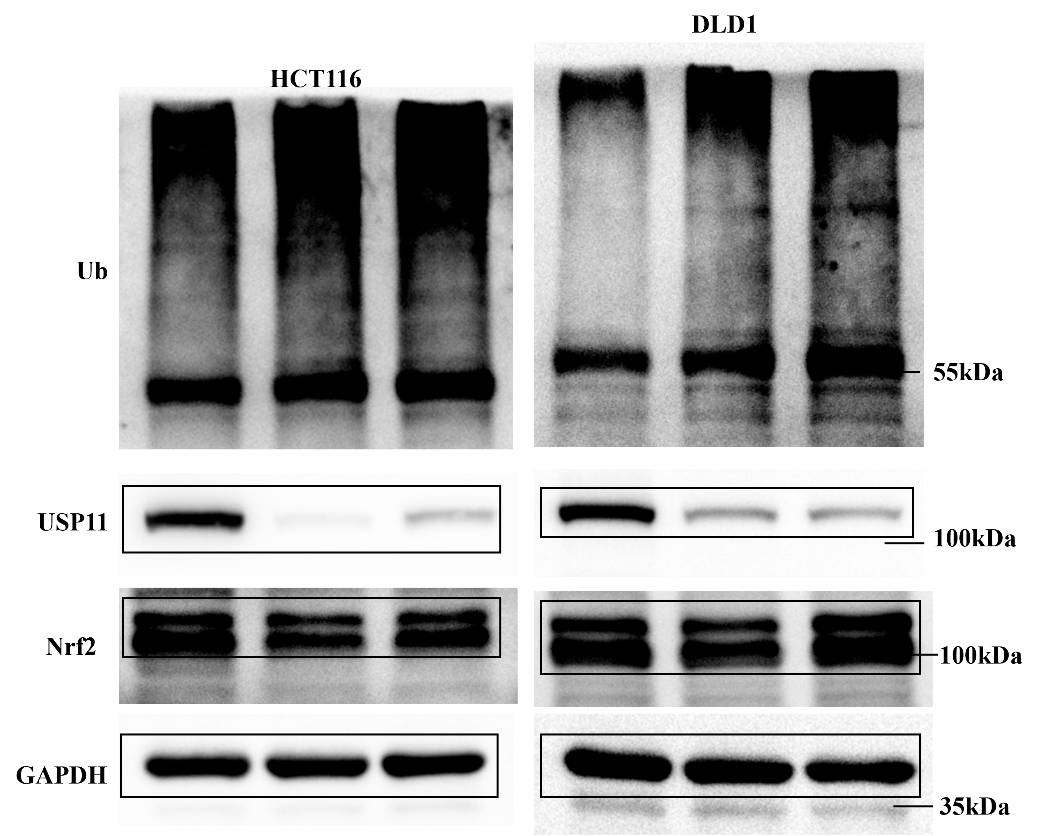


Figure4F


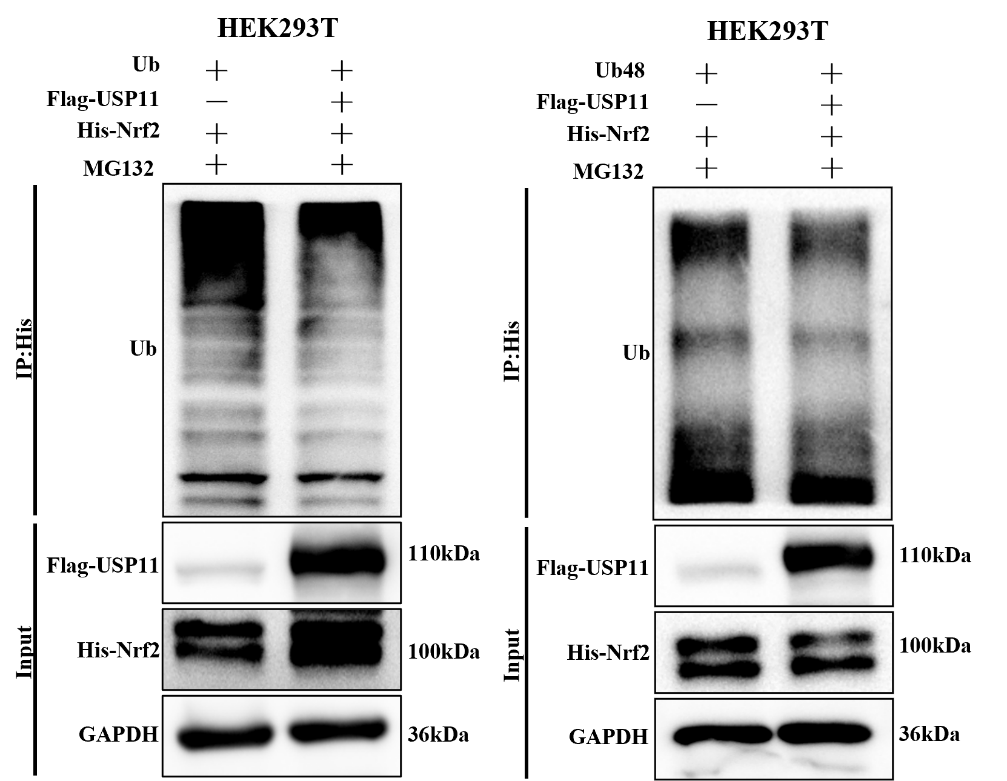


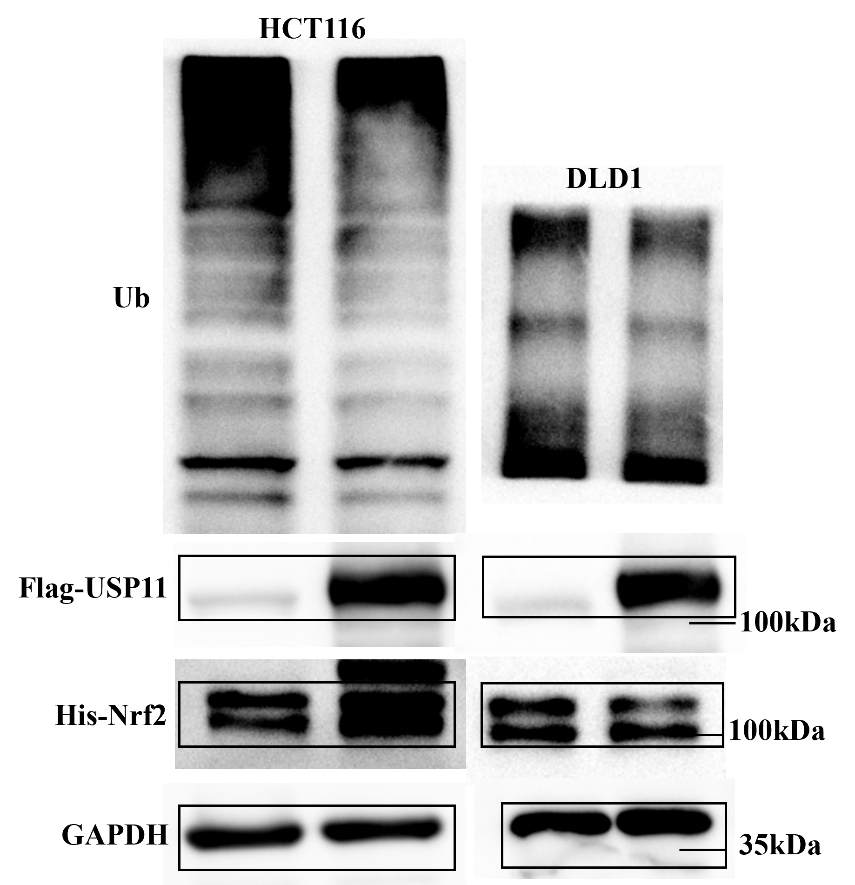


Figure6A


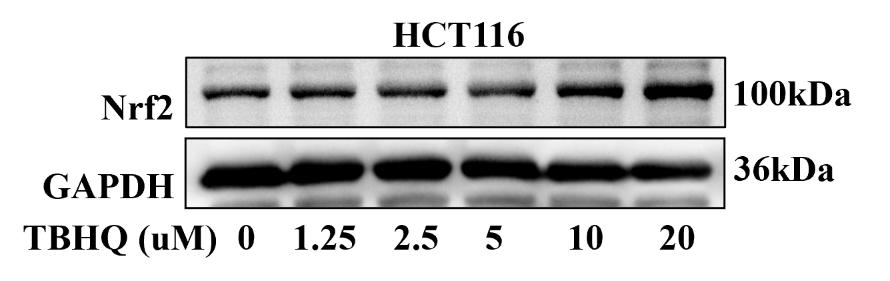


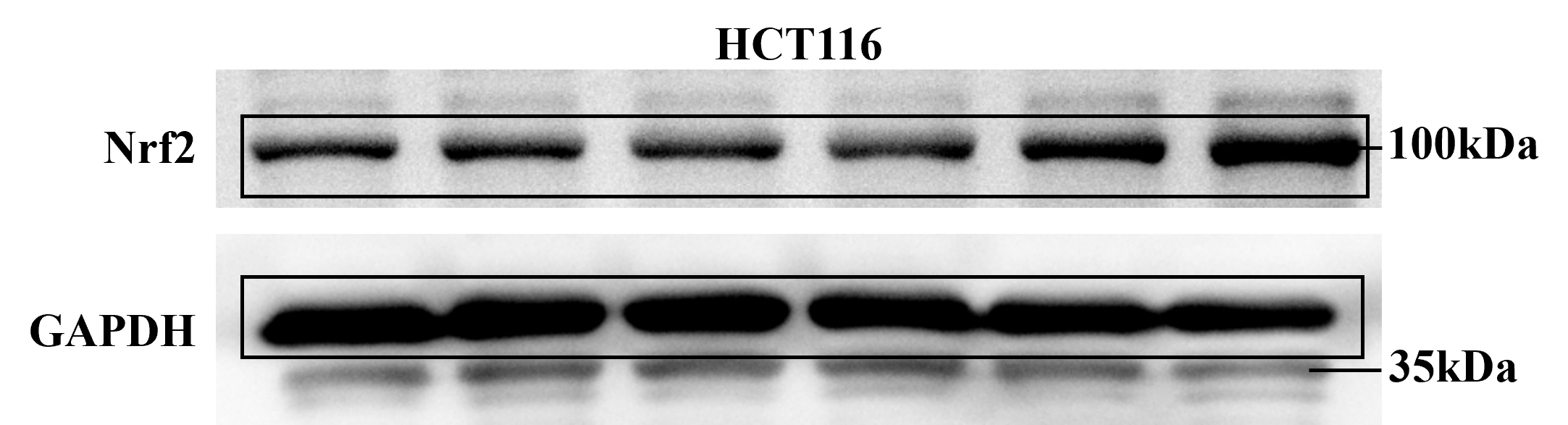


Figure6H


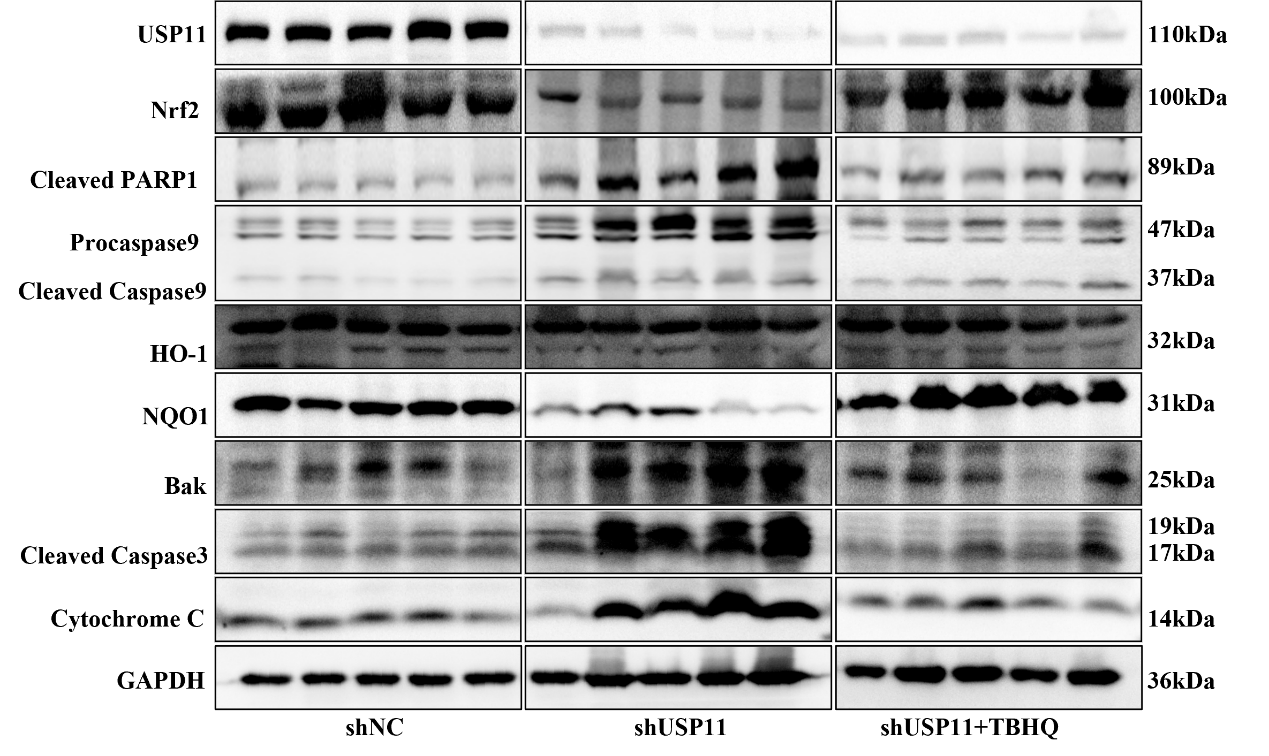


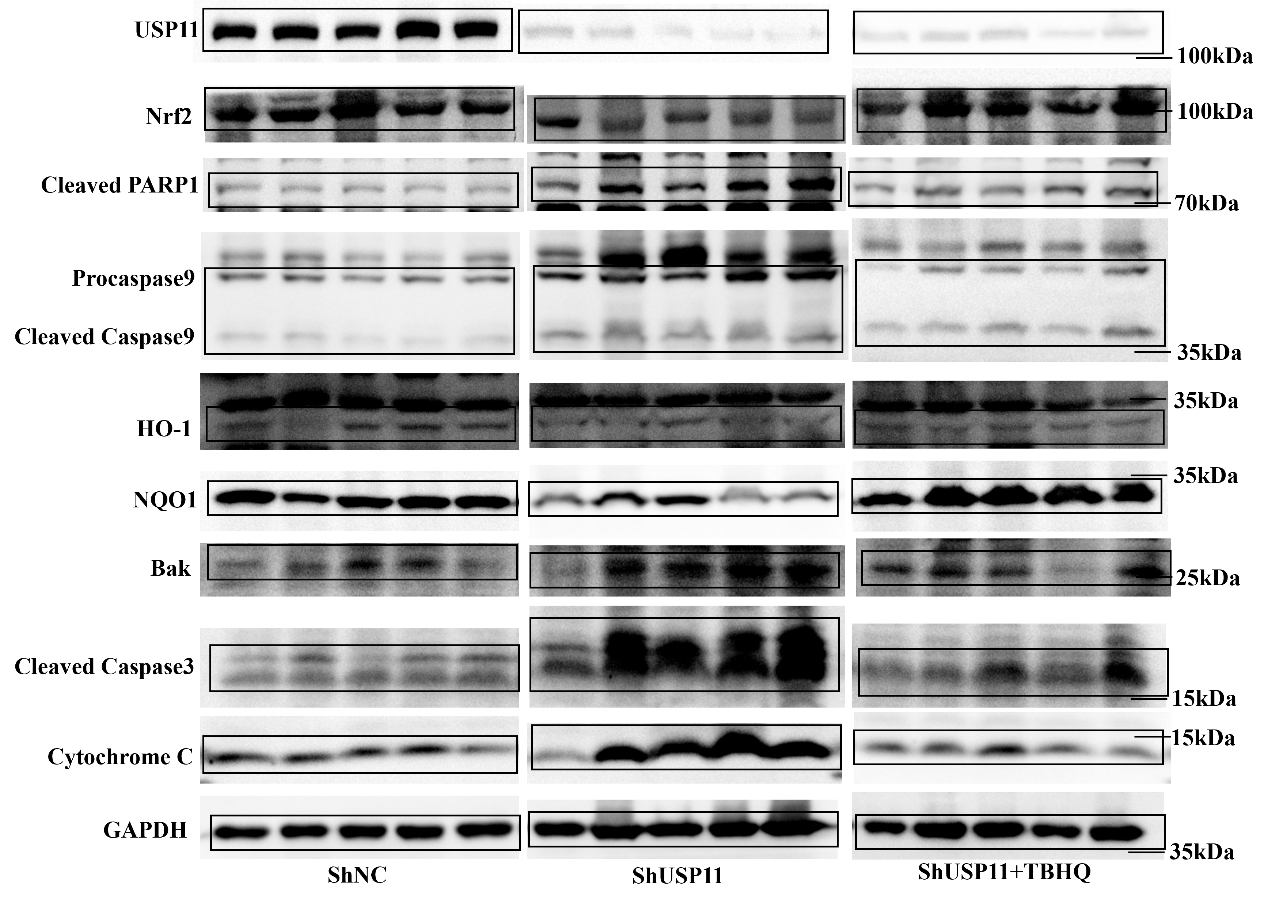


Figure7B


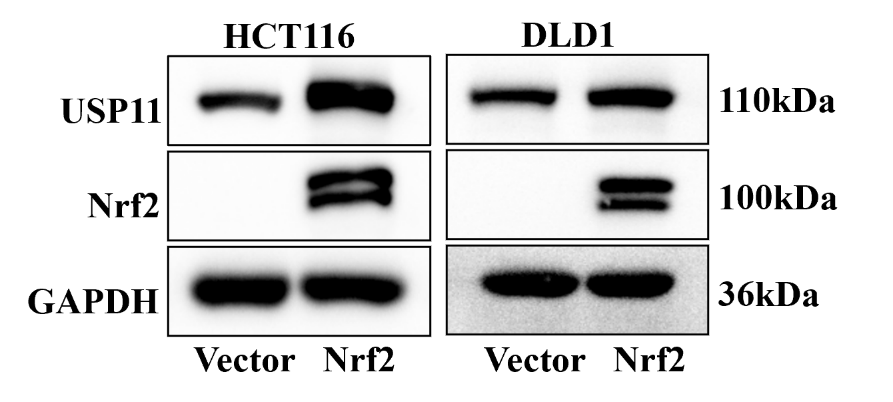


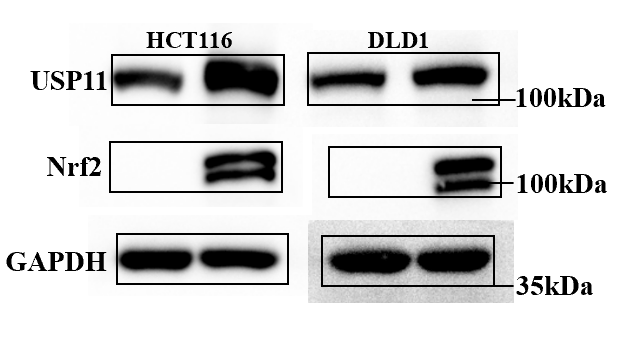


FigureS2B


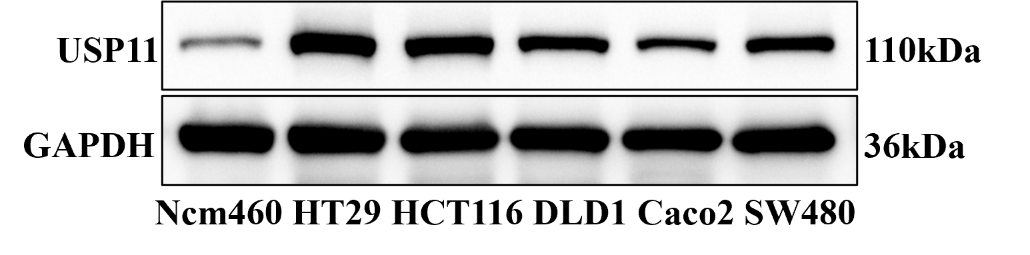


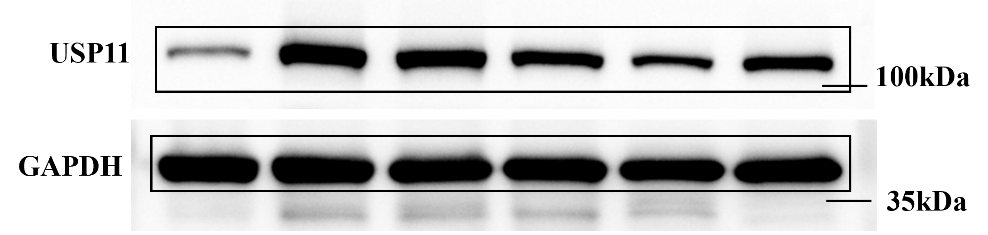


FigureS3A


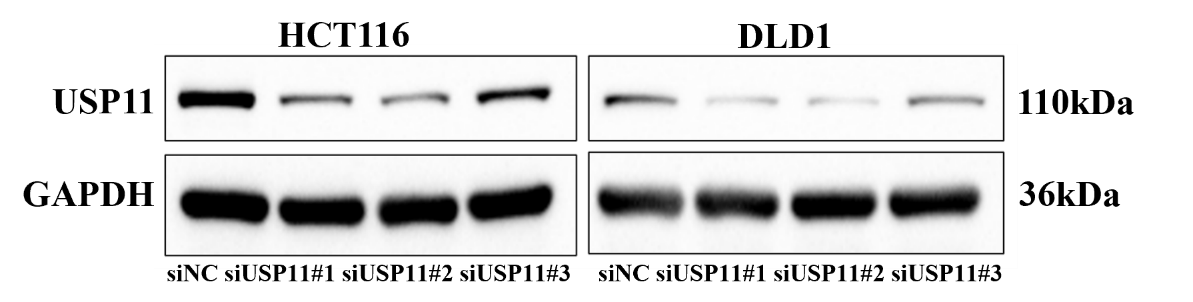


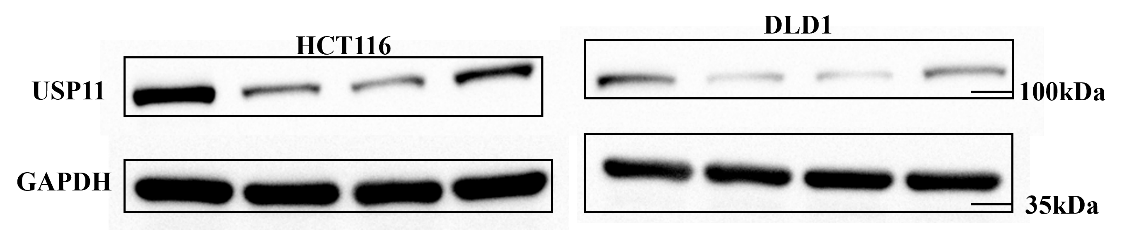


FigureS4C


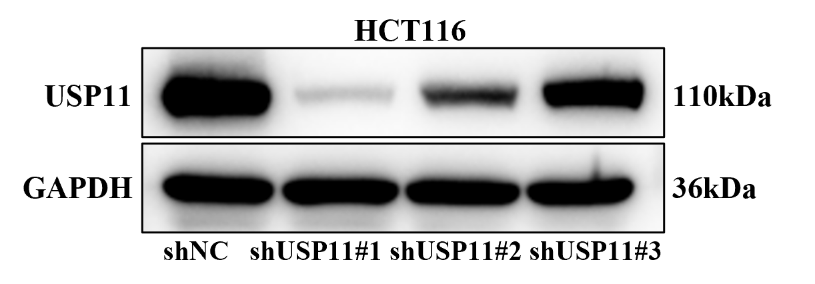


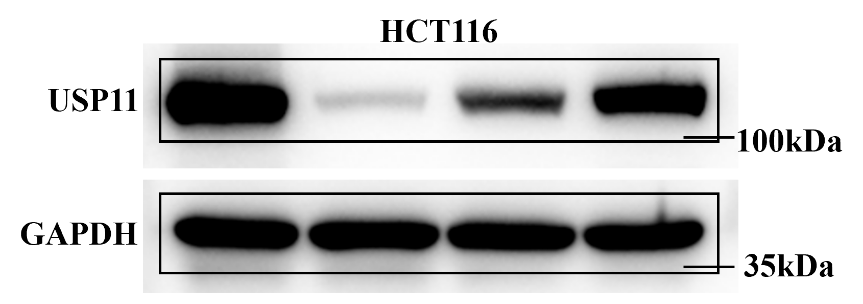

Supplement: Supplementary file 2 — Original Data [file 41419_2024_7188_MOESM2_ESM.docx]
